# Supplementary material for: A First-line management team’s strategies for sustaining resilience in a specialised intensive care unit—a qualitative observational study
Source: BMJ Open. 2021 Mar 15;11(3):e040358. doi: 10.1136/bmjopen-2020-040358 (PMC7970205; doi:10.1136/bmjopen-2020-040358)
Supplement: Supplementary data [file bmjopen-2020-040358supp001.pdf]

Appendix 1

Q1

(Clinical coordinator) We have put those two [patients] together in 9:1 to get an emergency cot in 9:2 for the twins [9 refers to a room divided in two sections equipped for one patient per section 9:1 and 9:2], I told the father that they may have to move out. But then we'll be in the situation where they maybe… They may need… That father is very new. So, I think that in that case they'll have to be two [staff] out there too.
(Operations manager) The [NN] twins?
(Head nurse) Right. Now there are two kids there, so one person can take care of them. But if you add those two [twins] then there'll be four babies, so then you need another person out there [on the ward] and there'll only be two staff left in there [room 9].
(Operations manager) Mm, it's tricky.
(Head nurse) Oh... so there are three of them who can help each other and… in [room] 7 and 8?
(Operations manager) Okay… I understand that to mean that we have a bit of leeway here if we need it in an emergency... but nothing that we can give to anyone else. Okay. [previously wanted to lend a nurse to another ward in need]
(Head nurse) Yeah, that's too bad. We'd love to be able to.
(Operations manager) Take over week 36 twins who need it, say. And then send them back, that's no fun.
(Clinical coordinator) And then we'd have to open like [rooms] 13 and 14.

Q2

The coordinator calls for an ad-hoc on-site face-to-face discussion with the strategic operations manager. This happens when the CMT experience that basic safe care practices at the clinical level consume every opportunity for maintaining overarching quality goals, the unit stop lending help to others and start sacrificing continuity of care for individual patients. The plan for today is to admit one intermediary level patient that were born during the night and is waiting for a room at the NICU. One patient has been diagnosed with a multi-resistant bacterium and will need increased hygiene standards. One baby currently in another hospital are being assessed by a surgeon for the need of possible transport and surgery, one will possibly arrive by helicopter for eye surgery today. Three babies are planned for being transported back to their respective local hospitals, one by helicopter today and twins tomorrow (Observers notes after the coordinating management team's morning huddle).

- *(Clinical coordinator) We are plenty of people today, that is nice. But when NN [strategic operations manager] asked if we had a lot of capacity, I had to say no.*

The clinical coordinator checks the antenatal list of at-risk mothers who are under observation.

- *(Clinical coordinator) Oh, there is a lot here, induction, ich... a lot of bleedings. There is a lot waiting... but you don't know when... It could be calm... I will not read all their charts.*
- *(Operations manager) ...one important thing. This baby that needed eye surgery is now acute and will arrive soon. They will land directly in the operating room and the transport team will take care of the baby until it can go back home its own hospital.*
- *(Strategic operations manager) Can we lend out some staff to [a neonatal ward in a neighbouring hospital].*
- *(Head nurse) I can check, we have plenty of staff, but they are all in use.*

- *(Strategic operations manager) I ask because they are not doing so well [the other unit]. Twins are being born and they are asking for allowance and resources to move them to another region.*

Q3

- *(Clinical coordinator) This is not good, its full [the ward]. We have no space when this eye baby arrives.*

The clinical coordinator walks to the room where a nurse oversees the twins that were supposed to be transported out the next day but are now showing symptoms of infection.

- *(Clinical coordinator) Lets´ see when [Strategic operations manager] gets back, maybe we can send someone else.*
- *(Nurse) But they went to fetch the week 31 from delivery to there [points to an empty cot].*
- *(Clinical coordinator) Yes, and the eye baby is coming [points toward the 2nd empty place for a cot. Then its full here. Do you think these twins could be together in a twin cot?*
- *(Nurse) Well, I don´t know. This one is just on the margin of managing without incubator, and that one is getting treatment for bilirubin [treatment includes being exposed to light from a special lamp].*
- *(Clinical coordinator) The problem is that I don't have staff to open another room.*
- *(Nurse) Maybe if we reconsider the lamp-treatment...*
- *(Clinical coordinator) Exactly, and then we hope that the eye baby can go back to his own hospital later in the evening.*
