# Supplementary material for: A First-line management team’s strategies for sustaining resilience in a specialised intensive care unit—a qualitative observational study
Source: BMJ Open. 2021 Mar 15;11(3):e040358. doi: 10.1136/bmjopen-2020-040358 (PMC7970205; doi:10.1136/bmjopen-2020-040358)
Supplement: Author's manuscript [file bmjopen-2020-040358.draft_revisions.pdf]

BMJ Open is committed to open peer review. As part of this commitment we make the peer review history of every article we publish publicly available.

When an article is published we post the peer reviewers' comments and the authors' responses online. We also post the versions of the paper that were used during peer review. These are the versions that the peer review comments apply to.

The versions of the paper that follow are the versions that were submitted during the peer review process. They are not the versions of record or the final published versions. They should not be cited or distributed as the published version of this manuscript.

BMJ Open is an open access journal and the full, final, typeset and author-corrected version of record of the manuscript is available on our site with no access controls, subscription charges or pay-per-view fees (http://bmjopen.bmj.com).

If you have any questions on BMJ Open's open peer review process please email info.bmjopen@bmj.com

A FIRST LINE MANAGEMENT TEAM´S STRATEGIES FOR SUSTAINING RESILIENCE IN A SPECIALISED INTENSIVE CARE UNIT – AN OBSERVATIONAL STUDY

Journal:	*BMJ Open*
Manuscript ID	bmjopen-2020-040358
Article Type:	Original research
Date Submitted by the Author:	11-May-2020
Complete List of Authors:	Hybinette, Karl; Karolinska Institutet Department of Learning Informatics Management and Ethic; Karolinska Hospital, Astrid Lindgrens Childrens Hospital<br>Pukk Härenstam, Karin; Karolinska Institutet Department of Learning Informatics Management and Ethic; Karolinska University Hospital, Paediatric Emergency Department<br>Ekstedt, Mirjam; Karolinska Institutet, LIME; Linneuniversitet,
Keywords:	QUALITATIVE RESEARCH, HEALTH SERVICES ADMINISTRATION & MANAGEMENT, Health policy < HEALTH SERVICES ADMINISTRATION & MANAGEMENT, Organisation of health services < HEALTH SERVICES ADMINISTRATION & MANAGEMENT, Risk management < HEALTH SERVICES ADMINISTRATION & MANAGEMENT

SCHOLARONE™ Manuscripts

I, the Submitting Author has the right to grant and does grant on behalf of all authors of the Work (as defined in the below author licence), an exclusive licence and/or a non-exclusive licence for contributions from authors who are: i) UK Crown employees; ii) where BMJ has agreed a CC-BY licence shall apply, and/or iii) in accordance with the terms applicable for US Federal Government officers or employees acting as part of their official duties; on a worldwide, perpetual, irrevocable, royalty-free basis to BMJ Publishing Group Ltd ("BMJ") its licensees and where the relevant Journal is co-owned by BMJ to the co-owners of the Journal, to publish the Work in this journal and any other BMJ products and to exploit all rights, as set out in our licence.

The Submitting Author accepts and understands that any supply made under these terms is made by BMJ to the Submitting Author unless you are acting as an employee on behalf of your employer or a postgraduate student of an affiliated institution which is paying any applicable article publishing charge ("APC") for Open Access articles. Where the Submitting Author wishes to make the Work available on an Open Access basis (and intends to pay the relevant APC), the terms of reuse of such Open Access shall be governed by a Creative Commons licence – details of these licences and which Creative Commons licence will apply to this Work are set out in our licence referred to above.

Other than as permitted in any relevant BMJ Author's Self Archiving Policies, I confirm this Work has not been accepted for publication elsewhere, is not being considered for publication elsewhere and does not duplicate material already published. I confirm all authors consent to publication of this Work and authorise the granting of this licence.

For peer review only

A FIRST LINE MANAGEMENT TEAM´S STRATEGIES FOR SUSTAINING RESILIENCE IN A SPECIALISED INTENSIVE CARE UNIT – AN OBSERVATIONAL STUDY

Authors

Karl Hybinette[1,2], Karin Pukk Härenstam[1-3], Mirjam Ekstedt[1, 4]

Affiliation

[1] Department of Learning, Informatics, Management and Ethics, Karolinska Institutet, Stockholm, Sweden

[2] Theme Children & Women´s Health, Karolinska University Hospital, Stockholm, Sweden

[3] Paediatric Emergency Department, Karolinska University Hospital, Stockholm, Sweden

[4] Department of Health and Caring Sciences, Linnaeus University, Kalmar, Sweden

Corresponding author

Karl Hybinette

Karolinska Institutet

Department of Learning, Informatics, Management and Ethics,

Tomtebodavägen 18 A

SE 171 77 Stockholm

E-mail: hybinette.karl@ki.se

Phone: 0046704297173

For peer review only

For peer review only - http://bmjopen.bmj.com/site/about/guidelines.xhtml

ABSTRACT

Objectives

Acute care units manage high risk patients at the limits of scientifically established treatments and organisational constraints aiming to balance reliability to standards with the needs of situational adaptation (resilience). First line managers have a central role of coordinating clinical care. Any systemic brittleness will be evident only in retrospect through for example care quality measures and accident statistics, which poses a challenge to understanding what successful managerial strategies for adaptation are and how they could be improved. The managerial work of balancing reliability and adaptation is only partially understood. This study aims to explore and describe how system resilience is sustained by naturally occurring coordination performed in situ by a management team under variable circumstances.

Design

An explorative observational study using shadowing observations of coordination analysed with inductive-deductive content analysis from a perspective of resilience engineering (RE).

Participants

A team of managers in a neonatal intensive care unit (i.e. clinical coordinators, head nurses, senior medical doctors).

Results

Managers focused on maintaining coherence in escalating problematic situations by facilitating teamwork through goalsetting, problem-solving and circumventing the technical systems' limitations in creating situational awareness. The results describe a functional relationship between the units' level of manoeuvrability and a progression of strategies for enabling adaptations for safe performance, expressed through recurring patterns of adaptation when the unit were subjected to pressure.

For peer review only - http://bmjopen.bmj.com/site/about/guidelines.xhtml

For peer review only

Conclusions

Coordination supports a coherent goal setting by increased team collaboration and is supported by team members ability to predict the behavior of each other. Our findings suggest that resilience is possible to engineer by supporting and training managers to maintain cohesion, manage system boundaries, adapting the structure and roles of the coordinating management team and shifting between information sources in response to the variability of the systems operating point (how tightly coupled the system is).

Keywords

Qualitative research, Health services administration & management, Health policy, Organisation of health services, Risk management

Wordcount

4417

Strengths and limitations of this study

- Several iterations of data collection and analysis allowed for an initially wide and later more focused data collection that opened opportunities for the researchers to follow up on specific findings.
- The explorative ethnographic design allowed for a deeper understanding of the underspecified (hidden) work of first line managers.
- This is a single center study of a specialised unit with a specific patient clientele that cannot be cared for by any other type of unit which might affect the generalizability and transferability of results.

For peer review only

For peer review only - http://bmjopen.bmj.com/site/about/guidelines.xhtml

66 • The first author (KH) who conducted the fieldwork is an experienced intensive care nurse. The familiarity with the specific type of work may have affected the interpretations drawn in this study.

For peer review only

For peer review only - http://bmjopen.bmj.com/site/about/guidelines.xhtml

BACKGROUND

The Neonatal Intensive Care Unit (NICU) continuously evolves by incorporating new care practises, organisational models and patient groups (1). It is a complex adaptive system (CAS) because of its many interrelated parts, goals and relationships where highly specialised care is fragmented over many places, purposes and teams of professionals (2). Work is done at the frontiers of scientifically established treatments, frequently pushing the organization to its limits (3). The difficulty of maintaining quality and safety is a persistent problem for everyday clinical work (ECW) in this high risk environment that is continuously exposed to acute admissions, staff shortage and rapidly deteriorating patients with life-threatening conditions (4,5).

Patient safety is often defined as an absence of certain outcomes such as 'the absence of preventable harm to a patient during the process of health care' (6) or 'the absence of the potential for, or the occurrence of, health care associated injury to patients, created by avoiding medical errors and taking action to prevent errors from causing injury' (7,8). However, during the last decades of patient safety research a movement have emerged towards an inclusive view of supporting the healthcare systems´ ability to sustain required (normal) operations complimentary to traditional error reduction and fault-finding, the resilience engineering (RE) perspective (9).

Resilience in healthcare is defined as 'the ability of the health care system (a clinic, a ward, a hospital, a country) to adjust its functioning prior to, during, or following events (changes, disturbances and opportunities), and thereby sustain required operations under both expected and unexpected conditions' [7, p. xvii]. An important point made within the RE perspective is that safety in ECW can be managed by a combination of structure and control on one hand (i.e. Safety -1) and adaptive behaviour on the other (Safety –2) (10).

For peer review only - http://bmjopen.bmj.com/site/about/guidelines.xhtml

Research on resilience in healthcare provides two important insights for how to enhance its capacity for resilient adaptations. Firstly, by making early investments in staff´s expertise, deep domain knowledge, clinical training and the maintenance of a good workplace culture and thus creating preconditions for teams to manoeuvre the system even under unexpected situations (11). Secondly is the understanding that successful as well as unsuccessful outcomes emerge from the fluid arrangement of system components (coordination) (12). Coordination is defined as "*…the deliberate and orderly alignment or adjustment of partners' actions to achieve jointly determined goals* (13). People who perform coordinative work are a part of a "*process by which team resources, activities, and responses are organized to ensure that tasks are integrated, synchronized, and completed within established temporal constraints"* (14)

Although coordinating functions are an integral part of everyday healthcare management in domains such as surgery, anesthesia, emergency and pediatric intensive care at the level of small multi-professional clinical teams (15–18), we know very little about how *managers* learn the strategies they use and which of the work practices they develop that are successful (19,20). Specifically multiteam coordination (i.e coordination within and between multiple teams) is not as empirically supported in highly specialised acute- or intensive care as in primary care with chronic patients (21) or teams in other high-risk domains such as aviation, military and maritime safety (22).

Therefore, it is important to further explore the performance of coordination within and between multiple teams in the acute care domain to allow for development of tools and training for supporting its managers.

This study aims to explore how system resilience is sustained by naturally occurring coordination performed in situ by a management team (i.e. clinical coordinators, head nurses, senior medical doctors) under variable circumstances.

For peer review only - http://bmjopen.bmj.com/site/about/guidelines.xhtml

METHODS

Design and setting

This explorative ethnographic study used participatory observations and an abductive approach to capture and analyse naturally occurring coordination in situ (23). The observations were conducted in a Swedish tertiary level NICU with an approximate capacity of 70 cots divided over three wards located in separate hospitals. The patient mix of the three wards is dependent on local factors such as the size and risk profile of adjacent delivery wards and the availability of paediatric surgical capacity. Staffing for the high acuity patients is normally one nursing team per two patients (one nurse and one assistant nurse). Paediatricians and neonatologists are allocated over the tree wards depending on availability and competence.

ECW in each of the three wards is managed by a clinical coordinator, a head nurse and an operations manager during daytime (Figure 1). The clinical coordinator performs tasks such as rostering, planning for patient flows (admissions, discharges and transports) and clinical work when needed. The head nurse is formally responsible for the work environment and quality of care. The operations manager is a senior neonatologist with an overall responsibility for the medical quality and patient flow. A strategic operations manager has mandate to move patients between hospitals within the own organisation or to hospitals outside. All managers are clinical specialists (nurses and neonatologists). Hereafter we will refer to this team as the coordinating management team (CMT).

[Insert figure 1 here]

Data collection

For peer review only - http://bmjopen.bmj.com/site/about/guidelines.xhtml

Data collection employed blended observational techniques including participatory observation of ‘places’, shadowing managers, ad-hoc interviews, collection of artefacts, field notes and memo writing (24). Observation of ‘places’ was primarily conducted in areas where the coordination of work was apparent, at the open office area where the head nurses and secretaries work and at the CMTs´ office at the center of the ward. Collected artefacts included coordinators own notes on patients´ statuses, occupancy- and rostering charts. All relevant aspects of the environment were captured in field notes during or after the observations, along with researchers’ memo-writing over personal reflections and thoughts about what was happening. The observer performed informal interviews with staff and managers during or after the shadowing. Clarifying questions were posed to get a broader picture of the observed situations. The reason for focusing on action-interaction was to capture a deep understanding of the varying conditions under which decisions and coordination happened (25).

Participant observations were conducted over the course of a year between 2016 and 2017. Beginning with a broad descriptive phase that later became focused (26). The focused observations took place during weekdays from 7 AM to 4 PM in January and February 2017. Starting with 50 hours of initial observations for the researcher to get familiar with the environment, staff and relevant aspects of the work for the subsequent observations. Four clinical coordinators were shadowed in their daily work for a total of six full shifts, meaning that the observer followed the clinical coordinator wherever he/she went (27,28). The observer attended both scheduled and ad hoc meetings between the managers and/or clinical staff (29), unless explicitly asked not to by any of the participants (30).

Data analysis

All meetings and interviews were audio recorded and transcribed verbatim. Transcriptions from tape-recorded dialogue were placed in a correct temporal order along with the field notes, so that the mix of field notes and transcription chronologically represented the full workday. The transcripts were read

For peer review only - http://bmjopen.bmj.com/site/about/guidelines.xhtml

through several times, followed by discussions in the research group about the level of detail in the data and reflections the observer had regarding the observed work shift (31). The initial inductive analysis went through a two-step process (columns two and three in Table 1). First, a conversation or a situation of relevance for the study's´ aim (a meaning unit) was selected and the question "What is happening here?" was directed to data. Next, the selected conversation or situation was analysed in the context of the entire scene where it took place, with the question "Why or how is it happening here?" The interpretation was condensed and labelled with one or several codes (Table 1). The initial inductive analysis generated a total of 86 codes. The codes and their relations were frequently discussed in the research team and sorted into tentative sub- and main categories. The movement back and forth between induction and deduction was a way to discover meaningful underlying patterns that made it possible to integrate concrete behaviour and deep contextual structures. The last iteration of analysis was a deductive comparison of interdependencies between the categories and sub-categories in relation to the theoretical concepts or resilience (25). Categories and subcategories were refined, and the researchers agreed upon six subcategories and two main categories that unified them.

For peer review only - http://bmjopen.bmj.com/site/about/guidelines.xhtml

Table 1 Example of the analysis in two-steps: going from *raw data* to *interpretation of incident* and to *analytical interpretation in context*, *code* and *memo*.

**Raw data**	**Interpretation of incident**	**Analytical interpretation in context**	**Code**	**Memo**
The observation begins in the flow room. The flow room is a small room with two workstations where the coordinator has her seat. There are several information sources hanging on the walls and post-it notes on the computers. The coordinator meets the observer in the flow room after having walked around the ward to check all the patient rooms.	In the physical environment, several different communication tools are gathered within a small area, i.e., tools used to summarize, remember, and disseminate important information. Information exchange occurs on paper notes stuck to computers, through software, and when the coordinator herself walks around the ward.	Information exchange is one part of coordination and can be performed through predefined channels and tools, but also more intuitively through physical meetings within the ward while the manager compares notes to what is experienced. The environment has been adapted for having several different information channels intersecting in one place.	Adapting environment – cluster tools for facilitating information exchange	The coordinator has gathered information about the current situation in the patient rooms by walking around and taking a look. She then looks at the system level, occupancy lists, and information from the other coordinators. If coordination means exercising control, real life information gathering is probably an important step.
The coordinator sits down at a computer and begins counting patients on her paper copy of the occupancy list and in TakeCare [a patient journal system]. She reports the occupancy in Belport and talks a bit about this.	Counting patients and manually entering the number into the national occupancy report is one of the first things the coordinator does in the morning.	The coordinator gets an idea of the status and distributes it to the rest of the country as information. This is proactive management, as future coordination may become easier if reference can be made to Belport, or if you know that the other units in the country your information.	Information handling	Reporting your status is a way of exercising control by impacting others perception regarding the degrees of freedom at your end.

PATIENT AND PUBLIC INVOLVMENT

173 Patients or the public were not directly involved in the development of this research. The researcher's involvement with clinical experts ensured that the patient perspective were in focus.

175 **RESULTS**

176 The NICU serves as its own emergency department, intensive care unit and a pre- and postoperative ward, requiring a wide array of interrelated multi-professional teams to operate in a coordinated fashion. The many roles of the unit are coupled with the pressures of maintaining high-quality and individualized care, family support and safe care practices. Some situations are manageable by reorganizing teams internally while others require the cooperation of external units (see Figure 1).

181 The ´how´ and ´when´ of coordination is presented in Table 2 that illustrate the functional relationship between the two main categories *adaptations for enabling safe performance* and *maneuverability* with their respective sub-categories. The sub-categories *Supporting system cohesion* and *Extending system boundaries* describe the joint work of managers and clinical staff. *Adapting the structure and roles of the coordinating management team* and *Shifting between information sources for better sensemaking* describe the management teams internal work.

187 *Extracts from the field notes and conversations during the participatory observations are presented to clarify the findings. More comprehensive material is attached in the appendix 1*

For peer review only - http://bmjopen.bmj.com/site/about/guidelines.xhtml

Table 2 The main categories are on the y-axis *adaptations for enabling safe performance* and on the x-axis; *maneuverability.* Four sub-categories on the y-axis describe strategies (i.e. the work that managers do); and the three sub-categories on the x-axis describe a progression of the strategies in relation to a perceived level of manoeuvrability.

		**Manoeuvrability**		
		**Losing control – reorganisation**	**Stretching the system to work outside ordinary conditions**	**Everyday work under ordinary conditions**
**Adaptations for enabling safe performance**	**Supporting system cohesion**	• Delay work and evaluate the situation<br>• Isolate problems and focus on re-creating manoeuvrability (creating systemic degrees of freedom)<br>• Exploit possibilities of extraordinary individual achievements (trade-off individual resilience for systemic control)	• Goalsetting towards protecting manoeuvrability (protecting systemic degrees of freedom)<br>• Goalsetting for promoting basic safe care practices at the clinical level through minimal staff allocation and skill mix<br>• Sacrificing continuity in patient assignments	• Goalsetting towards family-centred care<br>• Goalsetting of individualised care<br>• Managing optimal staff allocation for maintaining professional development and education<br>• Controlling occupancy and redundant capacity through predefined strategies<br>• Monitoring state of the ward at the clinical level by regular walkarounds in the clinical work environment
	**Extending system boundaries**	• Identifying novel use of any existing external resources (i.e. use of PETS transport team and other wards)<br>• Shedding managerial tasks for participating in clinical emergency work (trade-off management system for clinical system)	• Managing occupancy trade-offs between facilities and staffing (higher occupancy in fewer rooms lowers staff requirements).<br>• Utilising individual managers social networks within predefined limits for proactive problem-solving	• System working within normal boundaries
	**Adapting the structure and roles of the coordinating management team**	• Make *loss of control* explicit in the CMT.<br>• Moving from understanding the situation to making rapid decisions close to the clinical level	• Relaying information on the patients' clinical situation to mid- and upper-level managers (information priority bottom-up)	• Relaying high level plans to clinical level workers through regular briefings (information priority top-down)<br>• Participate in regular medical discussions on patients' status´
	**Shifting between information sources for better sensemaking**	• Dropping computerised aides, rostering systems and staffing charts for handwritten notes and memos<br>• Using face-to-face communication with people in close vicinity (shedding electronic communication)	• Seeking ad-hoc meetings within the CMT for calibrating information of the situation and possible workarounds<br>• Verbally explaining situations to other managers	• Regular use of computerised systems and manual cognitive aides

Manoeuvrability

The CMT work with limited staffing to achieve high occupancy while supporting clinical teams in their care of patients. They use clinical expertise to understand the overall situation and their management expertise to find a way forward as illustrated in the following quote.

"We have put those two [patients] together in 9:1 to get an emergency cot in 9:2 for the twins [9 refers to a room divided in two sections equipped for one patient per section 9:1 and 9:2], I told the father that

For peer review only

they may have to move out. But then we'll be in the situation where they maybe… They may need… That father is very new. So, I think that in that case they'll have to be two [staff] in there too (Clinical coordinator). [Q1 – appendix 1]

When several managers meet, they discuss the fit of different situations across the unit and adjust their strategies accordingly. Some priorities are accepted while others are discarded as unacceptable. This is described in x-axis of Table 2 as a progression of the sub-categories *Everyday work under ordinary conditions, Stretching the system to work outside ordinary conditions and Losing control – reorganisation.*

Everyday work under ordinary conditions

Coordination under ordinary conditions is largely based on preparations and routine. Even if one of the clinical teams are under heavy stress the situation is contained. The CMT have planned meetings, managers sweep the ward to collect up to date information on the state of things and relay high level plans to workers.

The coordinator begins her shift by conducting a walkaround of the ward, saying hello to the nurses and doctors as she is asking if everything is all right. After the walkaround she sits down to begin exploring her staff roster and patient occupancy charts (Observers notes).

Under ordinary conditions there is a minimal observable need for managers to manually adapt any information they extract from technical systems, regarding for example occupancy, patient acuity levels and staffing. Managers recognise weaknesses in the technical systems but can ignore them as they are not causing problems.

Stretching the system to work outside ordinary conditions

For peer review only - http://bmjopen.bmj.com/site/about/guidelines.xhtml

Some goals are not achievable as there is a lack of manoeuvrability for action to pursue them all. However, some can be sacrificed or be put on hold.

The following situation is a typical example of how the CMT must re-organize and adapt to out of ordinary conditions.

The coordinator calls for an ad-hoc on-site face-to-face discussion with the strategic operations manager. This happens when the CMT experience that basic safe care practices at the clinical level consume every opportunity for maintaining overarching quality goals, the unit stop lending help to others and start sacrificing continuity of care for individual patients. The plan for this day is to admit one intermediary level patient that were born during the night and is waiting for a room at the NICU. One patient has been diagnosed with a multi-resistant bacterium and will need increased hygiene standards (Observers notes) […] "We are plenty of people today, which is nice. But when NN [strategic operations manager] asked if we had a lot of capacity, I had to say no" (Clinical coordinator) […] "...one important thing. This baby that needed eye surgery is now acute and will arrive soon. They will land directly in the operating room and the transport team will take care of the baby until it can go back home its own hospital (Operations manager). [Q2 – Appendix 1]

The normal plan for postoperative care after acute eye-surgery is to be assigned a cot and a nursing team on the ward. The constrained situation is managed by using the transport team to temporarily care for the baby inside the NICU until the situation is stable enough for transport to another hospital. This decision has possible implications for the whole CMT. The clinical coordinator wants to know about the utilisation of staff and facilities. The head nurse about workplace safety, quality of care and economy. The operations manager has responsibility for the medical quality and strategic operations manager for the possibility of helping the other ward in trouble.

For peer review only - http://bmjopen.bmj.com/site/about/guidelines.xhtml

The ad-hoc meeting in this example illustrate that strategic goals of family centred care and optimal staff allocation can be traded for immediate medical priorities, basic safe care practises and protection of the wider system. Other patients on the ward can be safely tended to by their respective teams, unaware of this emerging crisis because it is handled by the transport team.

Losing control - reorganisation

The state of losing control is characterised by strategies for *sacrificing coordinative work tasks* and *focusing on re-creating manoeuvrability*. Now the focus shift to protecting clinical teams from being exposed to the rapidly shifting plans and priorities at the managerial level. Strategies for regaining control can be to sacrifice the goal of continuity by transferring at-risk mothers to other hospitals, or to temporarily transfer additional weight of medical care to neighbouring sub-systems or the CMT itself (the double headed arrows in Figure 1).

When the managers start shedding managerial tasks for doing operative work at the bedside, they will lose the ability to meet other managers and keep up to date with the ward. Management decisions in these situations are based on a narrower understanding of the bigger picture. Technical aides are less used (or not at all) because of their inability to present rapidly changing borderline conditions.

The following situation illustrates a reorganisation at the verge of losing control. The situation is that the coordinator tries to conserve an empty cot for possible acute emergency admissions.

"This is not good, its full [the ward]. We have no space when this eye baby arrives" (Clinical coordinator). The clinical coordinator walks to the room where a nurse oversees the twins that were supposed to be transported out the next day but are now showing symptoms of infection (Observers notes). "Do you think these twins could be together in a twin cot?" (Clinical coordinator) […] "Well, I don´t know. This one is just on the margin of managing without incubator" (Nurse) […] "The problem is that I don't have staff to open another room." (Clinical coordinator). [Q3 – Appendix 1]

For peer review only - http://bmjopen.bmj.com/site/about/guidelines.xhtml

The way to manage this situation is to concentrate available resources by putting twins together in one cot, using one nurse to care for three babies which is more than the goal of two babies per nurse. This manoeuvre creates an opportunity to temporarily have the capacity for five patients (including one empty emergency cot) in a room for four- and staffing for two patients. The nurse's expresses concern for her patient but remain focused on finding a solution.

Adaptations for enabling safe performance

Supporting system cohesion

Members of the CMT primarily communicate through ad-hoc meetings were verbal information is compared to lists of patients, patient conditions, staffing and workload indicators. All members of the CMT work close to the clinical context and use their up to date knowledge of the current goals' attainment for individual clinical teams and what the trade-offs are. With their experience of how things usually are, the managers compare computer- and paper-based cognitive aids, as well as the information provided at start-up meetings to update their understanding of the status of the ward and were it is heading (the umbrella perspective presented below in Figure 2). The CMT is constantly negotiating situations that need simultaneous attention, like prioritizing readiness and clinical capacity in some parts of the ward while maintaining family centered care and staff education in others.

Extending system boundaries

The need for extending the systems´ boundaries emerge as the pressure of prioritizing decisions increase. This allows for the CMT to make use of other units' facilities or staff, like delaying patients in the operating theatre or letting a transport team care for the patient for some time before handing it over to the units' regular staff. The CMT can use auxiliary staff and overlapping competencies of different professional groups, even managers can double as clinical staff within their vocation.

For peer review only - http://bmjopen.bmj.com/site/about/guidelines.xhtml

For peer review only

Adapting the structure and roles of the coordinating management team

The CMT express a range of strategies to fluently adapt its own structure in situations where there are not enough resources to manage within everyday routines, when there is urgency or when some of the CMT are not available with their specific expertise and mandate. Structural adaptation means that individuals in the CMT shift from relaying plans from top-down to working and gathering information from bottom-up.

Shifting between information sources for better sensemaking

Shifting information sources was observed when the CMT increased their reliance towards handwritten notes rather than the standard computer-generated lists for staffing and patient acuity information. Computer-generated aides work under normal conditions but does no longer serve their purposes as the situation on the ward become more complex.

Handwritten notes are normally used as memory aides in team discussions. The notes are mainly short phrases or single words in the margins like “discharge planned” or “need antibiotics”. These phrases carry specific meaning to the managers. The limitations of computer-generated lists to convey this type of information on real world complexities are expressed by members of the CMT. The national occupancy chart for example is only able to classify patients as high or low acuity. When there is a need to work outside the binary world of two patient groups, the team quit using this computer-generated aide and instead relied on their own domain knowledge, personal network and the stability of the CMT´s understanding of the big picture (Figure 2).

[Insert figure 2 here]

For peer review only - http://bmjopen.bmj.com/site/about/guidelines.xhtml

DISCUSSION

Our findings describe a link between adaptations for enabling safe performance and the available manoeuvrability. The CMT in this study exhibited a range of mindful adaptations for sustaining the units´ capacity for expressing resilience like sacrificing low level goals based on up-to date information and a continuous assessment of what would be minimally intrusive for the overall performance of the system (figure 2).

Supporting coherence

The CMT need to balance the demands and capacity of multiple teams that operate in separate rooms tending to patients with a wide variety of problems and acuity. Teams that for practical reasons not always meet to communicate with each other. A defining characteristic of a multiteam system is the ability of component teams to modify individual goal hierarchies while sharing a common distal goal or set of goals (20). Still, the CMT and the clinical staff consequently agree on making provision of acute care to rapidly deteriorating patients a top priority, allowing us to identify it as a core mission (i.e. purpose of the system) (32). Other priorities where more likely to be put on hold and resumed later or to be permanently dropped.

When the care teams are unable to communicate, an important factor for maintaining coherence was the managers maintenance of an umbrella perspective i.e. what was needed in order to understand how the bigger picture of their interventions fit together (33). At this point, it is possible to say two things about coherence that has implications for the trainability of managers as adaptive teams (34). First, that coordination supports a coherent goal setting with increased team collaboration and second, that it is enhanced by team members ability to predict the behaviour of each other.

For peer review only - http://bmjopen.bmj.com/site/about/guidelines.xhtml

Reorganising to support manoeuvrability

Everyday work of the CMT is characterised by seamlessly and actively organising and reorganising. The observations show how the CMT make use of early investments in for example staff´s expertise, deep domain knowledge and the workplace culture to maintain a unit wide focus on the core mission (11). Allowing the care teams to adapt their goal hierarchies individually exemplifies that being resilient is to be part of a process of identifying conflicting goals in a complex, intractable environment using *"numerous indicators in a proactive fashion to probe a system's adaptive capacity before system-wide collapse results in disaster"* (34). A realisation of studying the CMT was the shapelessness of the organisation. We could not observe a formal agenda for how and why the CMT was supposed to prioritize in terms of goal achievement below the core mission. Our study suggests that it is up to the CMT to support the system by using experience, professional ethos and domain knowledge to negotiate the way forward. Figure two illustrate one instantiation of this balancing act.

Important for the application of resilience in ECW is the link between adaptation and outcome (successful or unsuccessful) as described in for example the CARE model for researching resilience in healthcare (35). An adaptation is a deviation from work as planned, and it is not always clear beforehand whether the outcome of an adaptation constitutes success or failure in terms of quality and safety (a.a). Our study describe coordinating managers' adaptive responses to the conflicting demands of acute patient care on one hand and the long-term demands for quality and safety on the other (measured as for example respirator days, patient throughput and hospital acquired bloodstream infections). Assessment and subsequent learning from adaptive managerial responses to situations where the system is "stretched" or "needs reorganization" (table 2), should be about whether the actions were able to achieve the short-term goals while preserving the long-term goals of safe, high quality patient care.

For peer review only - http://bmjopen.bmj.com/site/about/guidelines.xhtml

Balancing between long- and short-term goals

Resilience is dependent on the use of earlier investments in potential opportunities for action described as systemic degrees of freedom (SDOF) (11). However, low-level goal-sacrifices represent a potential loss of future investments in SDOF if they are overexploited. For example, families will be less prepared to go home if they are not trained, staff might receive less time learning from experienced colleagues and formal routines might erode if they are not employed.

The balancing act of seemingly irreconcilable goals makes it impossible to decide in retrospect whether coordination has been good or bad for the total outcome of the system. Each decision to suspend or sacrifice a low-level goal has implications for the organisations future capacity for expressing resilience. All teams are working towards the core mission of providing acute care. But the maintenance of long-term investments is achieved by managing a diversity of low-level goal-sacrifices between many clinical teams (i.e. sacrificing patient education in one and staff education in another). For example, in Figure 2 the teams 1 and 2 can achieve parent education and family centred care because the third team can be isolated and focus on acute care.

CONCLUSIONS

only

We are only beginning to understand teams of managers strategies for prioritising and acting on the variability of SDOF (36). Task management, for example during medical resuscitation have well established decision support based on understanding of the human body. There is also a well-established body of knowledge around non-technical skills for clinical teams´ efficient teamwork during stabilisation of a critically ill patient (37). Our findings suggest that managers at the clinical level, while being central to the system’s capacity for expressing resilience, do not have a similar explicit model or

For peer review only - http://bmjopen.bmj.com/site/about/guidelines.xhtml

training for how they approach their work. Furthermore, managers lack the aid of tailored decision support systems. This could depend on well described challenges for design of such interventions (36). It is important that healthcare policy and organisational redesign initiated by a higher system level are well calibrated with the nature of managerial work on the clinical level before interventions can be developed.

Seeing that many healthcare systems are facing increasing resource constraints, resilience and sustainability becomes increasingly important. Our recommendations for future research and policy is to prioritise the study and operationalisation of successful coordination in a wide variety of contexts. For example, of how managers in the clinical context continually adapt instances of optimal staff allocation for maintaining professional development and education with promoting basic safe care practices at the clinical level (Figure 2).

ARTICLE SUMMARY

Limitations

This is a single centre study of a specialised unit with a specific patient clientele that cannot be cared for by any other type of unit available. It was expected that the unit´s high tempo and specialisation would promote a particularly observable coordinative work although it might introduce the argument of limited transferability to other areas of healthcare. Although the specific unit for study enabled us to capture and understand the subtleties of everyday work of first line managers, further studies are needed to investigate how much of this work that may be specific to organizations of both similar and contrasting types.

The first author (KH) who conducted the fieldwork is an experienced intensive care nurse. The familiarity with the specific type work may have affected the interpretations drawn in this study. Table 1 of the

For peer review only - http://bmjopen.bmj.com/site/about/guidelines.xhtml

methodological section provide a trail of how interpretations of data where made. Trustworthiness during data analysis was addressed by regular peer-check and in seminars with the wider research group (38). The iterative process of data collection and analysis was intended to secure that the analysis included more than one researcher's interpretation. Transferability were addressed by leaving an audit trail of extracts from the raw data in the report so that readers from other fields can evaluate if the results are transferable to their respective contexts (39).

For peer review only

LIST OF ABBREVIATIONS

RE: Resilience Engineering; NICU: Neonatal Intensive Care Unit; CAS: Complex Adaptive System; ECW: Everyday Clinical Work; CMT: Coordination Management Team; SDOF: Systemic Degrees of Freedom

DECLARATIONS

Ethics approval

This study was approved by the regional ethical review board of Stockholm (2016/1832-32).

Consent for publication

Not applicable.

Availability of data and material

Given that the data is in Swedish and phrasing of the consent obtained from participants, complete raw data are not available for sharing. Partial datasets used and/or analysed during the current study are available from the corresponding author on reasonable request. A selection of translated quotes is supplied in the results and methods sections and in appendix 1.

For peer review only - http://bmjopen.bmj.com/site/about/guidelines.xhtml

Competing interests

The authors declare that they have no competing interests.

Funding

The authors received no specific funding for this work.

Author Contributions

The study was initiated by KH. The study design was developed in collaboration within the research team. KH performed the data collection. The analysis and interpretation of data was conducted in close collaboration between KH, ME and KPH. KH wrote the first draft of the paper. All authors were involved in writing, critical revisions and approval of the final version.

Acknowledgement

Thank to Professor Eric Hollnagel that contributed with theoretical expertise on the interpretation of data; Professor Richard Cook for valuable discussions about system safety and methodology in the early phase of the study. Thanks also to the participating Neonatal clinic.

References

1. Braithwaite J, Churruca K, Ellis LA, Long J, Clay-williams R, Damen N, et al. Complexity Science in Healthcare – Aspirations, Approaches, Applications and Accomplishments: A White Paper. Australian Institute of Health Innovation, Macquarie University. Sydney; 2017.

2. Cilliers P. Complexity and postmodernism Understanding complex systems. Faraday Discussions. London: Routledge; 2002. 168 p.

3. Cook R, Rasmussen J. “Going solid”: A model of system dynamics and consequences for patient

For peer review only

safety. Qual Saf Heal Care. 2005;14(2):130–4.

4. Samra HA, McGrath JM, Rollins W. Patient safety in the NICU: A comprehensive review. J Perinat Neonatal Nurs. 2011;25(2):123–32.

5. Bondurant PG, Nielsen-farrell J, Armstrong L. The Journey to High Reliability in the NICU. J perinaltal Nurs. 2015;29(2):170–8.

6. World Health Organization. What is Patient Safety? [Internet]. 2018 [cited 2019 May 13]. Available from: http://www.who.int/patientsafety/about/en/ index.html

7. Committee on Data Standards for Patient Safety. Patient Safety: Achieving a New Standard for Care [Internet]. 2004. 550 p. Available from: http://www.nap.edu/catalog/10863.html

8. Network PS. Agency for Healthcare Research and Quality [Internet]. [cited 2018 Sep 10]. Available from: https://psnet.ahrq.gov/glossary/P

9. Wears RL, Hollnagel E, Braithwaite J. Resilient Health Care, Volume 2 : The resilience of everyday clinical work. Farnham: Ashgate Publishing Limited; 2015. 328 p.

10. Eurocontrol. From Safety-I to Safety-II: A White Paper. Netw Manag. 2013;1–32.

11. Ekstedt M, Cook RI. The stockholm blizzard of 2012. In: Resilient Health Care, Volume 2: The Resilience of Everyday Clinical Work. 2017. p. 59–74.

12. Cook RI. Taking Things in One's Stride: Cognitive Features of Two Resilient Performances. In: Hollnagel E, Woods DD, Leveson N, editors. Resilience Engineering: Concepts and Precepts [Internet]. Burlington: Ashgate; 2006. p. 205–21. Available from: http://qualitysafety.bmj.com/lookup/doi/10.1136/qshc.2006.018390

13. Gulati R, Wohlgezogen F, Zhelyazkov P. The Two Facets of Collaboration: Cooperation and

For peer review only

For peer review only - http://bmjopen.bmj.com/site/about/guidelines.xhtml

Coordination in Strategic Alliances. Acad Manag Ann [Internet]. 2012;6(1):531–83. Available from: http://search.ebscohost.com/login.aspx?direct=true&db=bth&AN=77495192&site=ehost-live&scope=site

14. Cannon-Bowers J, Tannenbaum S, Salas E, Volpe C. Defining team competencies: Implications for training requirements and strategies. In: Guzzo R, Salas E, Associates, editors. Team effectiveness and decision making in organizations. San Francisco, CA: Jossey-Bass; 1995. p. 333–80.

15. Bogdanovic J, Perry J, Guggenheim M, Manser T. Adaptive coordination in surgical teams: An interview study. BMC Health Serv Res. 2015;15(1):1–12.

16. Burtscher MJ, Wacker J, Grote G, Manser T. Managing nonroutine events in anesthesia: The role of adaptive coordination. Hum Factors. 2010;52(2):282–94.

17. Nugus P, Forero R, McCarthy S, Mcdonnell G, Travaglia J, Hilman K, et al. The emergency department "carousel": An ethnographically-derived model of the dynamics of patient flow. Int Emerg Nurs [Internet]. 2014;22(1):3–9. Available from: http://dx.doi.org/10.1016/j.ienj.2013.01.001

18. Douglas S, Cartmill R, Brown R, Hoonakker P, Slagle J, Schultz Van Roy K, et al. The work of adult and pediatric intensive care unit nurses. Nurs Res. 2013;62(1):50–8.

19. Cook R, Woods D. The messy details: Insights from technical work studies in healthcare. In: Proceedings of the human factors and ergonomics society 47th annual meeting. 2003. p. 2002–3.

20. Mathieu J, Marks MA. Multiteam Systems. In: Anderson N, Ones D, Sinangil HK VC, editor. Handbook of Industrial, Work & Organizational Psychology. London, England: SAGE Publications; 2001. p. 289–313.

21. Weaver S, Che X, Petersen L, Hysong S. Unpacking Care Coordination Through a Multiteam

For peer review only - http://bmjopen.bmj.com/site/about/guidelines.xhtml

System Lens: A Conceptual Framework and Systematic Review. Med Care [Internet]. 2018;56(3):247–59. Available from: http://ovidsp.ovid.com/ovidweb.cgi?T=JS&PAGE=reference&D=ovftt&NEWS=N&AN=00005650-201803000-00009

22. Shuffler ML, Jiménez-Rodríguez M, Kramer WS. The Science of Multiteam Systems: A Review and Future Research Agenda. Small Gr Res. 2015;46(6):659–99.

23. Silverman D. Interpreting Qualitative Data: Methods for Analysing Talk, Text and Interaction. 2 nd. London, England: SAGE Publications; 2001. 325 p.

24. Graneheim UH, Lindgren B, Lundman B. Methodological challenges in qualitative content analysis : A discussion paper. Nurse Educ Today [Internet]. 2017;56(June):29–34. Available from: http://dx.doi.org/10.1016/j.nedt.2017.06.002

25. Dubois A, Gadde LE. Systematic combining: An abductive approach to case research. J Bus Res. 2002;55(7):553–60.

26. Spradley JP. Participant observation. New York: Holt, Rinehart and Winston; 1980. 195 p.

27. Tengblad S. The Work of Managers: Towards a Practice Theory of Management [Internet]. Tengblad S, editor. The Work of Managers: Towards a Practice Theory of Management. Oxford University Press; 2012. 1–384 p. Available from: http://www.oxfordscholarship.com/view/10.1093/acprof:oso/9780199639724.001.0001/acprof-9780199639724

28. Arman R, Vie OE, Åsvoll H. Refining shadowing methods for studying managerial work. In: The Work of ManagersTowards a Practice Theory of Management [Internet]. Oxford University Press; 2012. p. 301–17. Available from:

For peer review only

For peer review only - http://bmjopen.bmj.com/site/about/guidelines.xhtml

http://www.oxfordscholarship.com/view/10.1093/acprof:oso/9780199639724.001.0001/acprof-9780199639724-chapter-16

29. Roth EM, Patterson ES. Using Observational Study as a Tool for Discovery: Uncovering Cognitive and Collaborative Demands and Adaptive strategies. In: Henry Montgomery, Raanan Lipshitz BB, editor. How Professionals Make Decision Volume 5. 5th ed. 2005. p. 379–93.

30. Johnson B. Ethical issues in shadowing research. Qual Res Organ Manag An Int J [Internet]. 2014;9(1):21–40. Available from: http://www.emeraldinsight.com/doi/10.1108/QROM-09-2012-1099

31. Strauss A, Corbin J. Basics Of Qualitative Research: Techniques And Procedures For Developing Grounded Theory. 4 th ed. London: SAGE Publications; 1998. 456 p.

32. Vicente KJ. Cognitive work analysis : toward safe, productive, and healthy computer-based work. London, England: Lawrence Erlbaum Associates; 1999. xix, 392 p.

33. Goldenhar LM, Brady PW, Sutcliffe KM, Muething SE. Huddling for high reliability and situation awareness. BMJ Qual Saf. 2013;22(11):899–906.

34. Gorman JC, Cooke NJ, Amazeen PG. Training adaptive teams. Hum Factors. 2010;52(2):295–307.

35. Anderson JE, Ross AJ, Jaye P. Modelling Resilience and Researching the Gap between Work-as-Imagined and Work-as-Done. In: J. Braithwaite, R. L. Wears RL and EH, editor. Resilient Health Care, Volume 3, Reconciling Work-as-Imagined and Work-as-Done. Farnham: Ashgate; 2017. p. 133–41.

36. Praetorius G, Hollnagel E, Dahlman J. Modelling Vessel Traffic Service to understand resilience in everyday operations. Reliab Eng Syst Saf. 2015;141:10–21.

For peer review only

For peer review only - http://bmjopen.bmj.com/site/about/guidelines.xhtml

37. Manser T. Teamwork and patient safety in dynamic domains of healthcare: A review of the literature. Acta Anaesthesiol Scand. 2009;53(2):143–51.

38. Lincoln YS. Emerging Criteria for Quality in Qualitative and Interpretive Research. Qual Inq [Internet]. 1995 Sep 29;1(3):275–89. Available from: http://methods.sagepub.com/book/the-qualitative-inquiry-reader/n19.xml

39. Elo S, Kääriäinen M, Kanste O, Pölkki T, Utriainen K, Kyngäs H. Qualitative Content Analysis. SAGE Open. 2014;4(1):215824401452263.

For peer review only

For peer review only - http://bmjopen.bmj.com/site/about/guidelines.xhtml

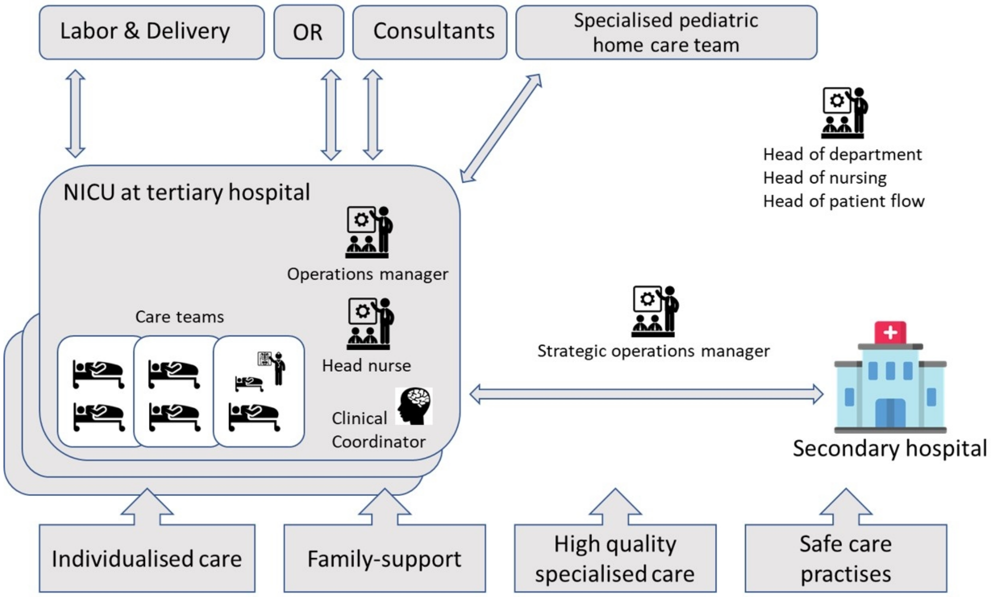

Fig. 1 The studied NICU in a tertiary level hospital with surgical capacity. Managers are located to illustrate their vicinity to the clinical care teams. Overarching goals are presented at the base of the figure. A selection of peripheral wards and units are illustrated in the surrounding area. The arrows are double headed to symbolise a two-way relationship of demands and possibilities for negotiation of for example patient transfers.

214x129mm (149 x 149 DPI)

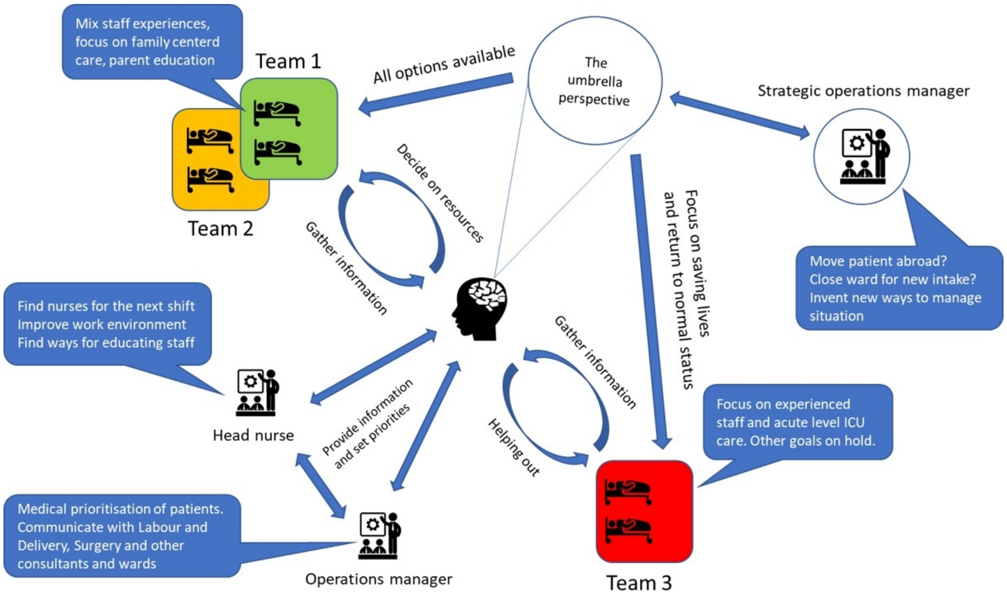

Fig. 2 The clinical coordinator at the centre of the CMT. The work of balancing goal-settings for system wide coherence, maintaining the umbrella perspective and adapting the CMTs´ work to meet rapidly changing demands.

204x124mm (149 x 149 DPI)

Appendix 1

Q1

(Clinical coordinator) We have put those two [patients] together in 9:1 to get an emergency cot in 9:2 for the twins [9 refers to a room divided in two sections equipped for one patient per section 9:1 and 9:2], I told the father that they may have to move out. But then we'll be in the situation where they maybe… They may need… That father is very new. So, I think that in that case they'll have to be two [staff] out there too.
(Operations manager) The [NN] twins?
(Head nurse) Right. Now there are two kids there, so one person can take care of them. But if you add those two [twins] then there'll be four babies, so then you need another person out there [on the ward] and there'll only be two staff left in there [room 9].
(Operations manager) Mm, it's tricky.
(Head nurse) Oh... so there are three of them who can help each other and… in [room] 7 and 8?
(Operations manager) Okay… I understand that to mean that we have a bit of leeway here if we need it in an emergency... but nothing that we can give to anyone else. Okay. [previously wanted to lend a nurse to another ward in need]
(Head nurse) Yeah, that's too bad. We'd love to be able to.
(Operations manager) Take over week 36 twins who need it, say. And then send them back, that's no fun.
(Clinical coordinator) And then we'd have to open like [rooms] 13 and 14.

Q2

The coordinator calls for an ad-hoc on-site face-to-face discussion with the strategic operations manager. This happens when the CMT experience that basic safe care practices at the clinical level consume every opportunity for maintaining overarching quality goals, the unit stop lending help to others and start sacrificing continuity of care for individual patients. The plan for today is to admit one intermediary level patient that were born during the night and is waiting for a room at the NICU. One patient has been diagnosed with a multi-resistant bacterium and will need increased hygiene standards. One baby currently in another hospital are being assessed by a surgeon for the need of possible transport and surgery, one will possibly arrive by helicopter for eye surgery today. Three babies are planned for being transported back to their respective local hospitals, one by helicopter today and twins tomorrow (Observers notes after the coordinating management team's morning huddle).

- *(Clinical coordinator) We are plenty of people today, that is nice. But when NN [strategic operations manager] asked if we had a lot of capacity, I had to say no.*

The clinical coordinator checks the antenatal list of at-risk mothers who are under observation.

- *(Clinical coordinator) Oh, there is a lot here, induction, ich... a lot of bleedings. There is a lot waiting... but you don't know when... It could be calm... I will not read all their charts.*
- *(Operations manager) ...one important thing. This baby that needed eye surgery is now acute and will arrive soon. They will land directly in the operating room and the transport team will take care of the baby until it can go back home its own hospital.*
- *(Strategic operations manager) Can we lend out some staff to [a neonatal ward in a neighbouring hospital].*
- *(Head nurse) I can check, we have plenty of staff, but they are all in use.*

For peer review only

- *(Strategic operations manager) I ask because they are not doing so well [the other unit]. Twins are being born and they are asking for allowance and resources to move them to another region.*

Q3

- *(Clinical coordinator) This is not good, its full [the ward]. We have no space when this eye baby arrives.*

The clinical coordinator walks to the room where a nurse oversees the twins that were supposed to be transported out the next day but are now showing symptoms of infection.

- *(Clinical coordinator) Lets´ see when [Strategic operations manager] gets back, maybe we can send someone else.*
- *(Nurse) But they went to fetch the week 31 from delivery to there [points to an empty cot].*
- *(Clinical coordinator) Yes, and the eye baby is coming [points toward the 2nd empty place for a cot. Then its full here. Do you think these twins could be together in a twin cot?*
- *(Nurse) Well, I don´t know. This one is just on the margin of managing without incubator, and that one is getting treatment for bilirubin [treatment includes being exposed to light from a special lamp].*
- *(Clinical coordinator) The problem is that I don't have staff to open another room.*
- *(Nurse) Maybe if we reconsider the lamp-treatment...*
- *(Clinical coordinator) Exactly, and then we hope that the eye baby can go back to his own hospital later in the evening.*

For peer review only

BMJ Open

A FIRST LINE MANAGEMENT TEAM´S STRATEGIES FOR SUSTAINING RESILIENCE IN A SPECIALISED INTENSIVE CARE UNIT – A QUALITATIVE OBSERVATIONAL STUDY

Journal:	*BMJ Open*
Manuscript ID	bmjopen-2020-040358.R1
Article Type:	Original research
Date Submitted by the Author:	03-Sep-2020
Complete List of Authors:	Hybinette, Karl; Karolinska Institutet Department of Learning Informatics Management and Ethic; Karolinska Hospital, Astrid Lindgrens Childrens Hospital<br>Pukk Härenstam, Karin; Karolinska Institutet Department of Learning Informatics Management and Ethic; Karolinska University Hospital, Paediatric Emergency Department<br>Ekstedt, Mirjam; Karolinska Institutet, LIME; Linneuniversitet,
**Primary Subject Heading**:	Medical management
Secondary Subject Heading:	Emergency medicine, Health policy, Health services research, Health informatics, Intensive care
Keywords:	QUALITATIVE RESEARCH, HEALTH SERVICES ADMINISTRATION & MANAGEMENT, Health policy < HEALTH SERVICES ADMINISTRATION & MANAGEMENT, Organisation of health services < HEALTH SERVICES ADMINISTRATION & MANAGEMENT, Risk management < HEALTH SERVICES ADMINISTRATION & MANAGEMENT

SCHOLARONE™
Manuscripts

I, the Submitting Author has the right to grant and does grant on behalf of all authors of the Work (as defined in the below author licence), an exclusive licence and/or a non-exclusive licence for contributions from authors who are: i) UK Crown employees; ii) where BMJ has agreed a CC-BY licence shall apply, and/or iii) in accordance with the terms applicable for US Federal Government officers or employees acting as part of their official duties; on a worldwide, perpetual, irrevocable, royalty-free basis to BMJ Publishing Group Ltd ("BMJ") its licensees and where the relevant Journal is co-owned by BMJ to the co-owners of the Journal, to publish the Work in this journal and any other BMJ products and to exploit all rights, as set out in our licence.

The Submitting Author accepts and understands that any supply made under these terms is made by BMJ to the Submitting Author unless you are acting as an employee on behalf of your employer or a postgraduate student of an affiliated institution which is paying any applicable article publishing charge ("APC") for Open Access articles. Where the Submitting Author wishes to make the Work available on an Open Access basis (and intends to pay the relevant APC), the terms of reuse of such Open Access shall be governed by a Creative Commons licence – details of these licences and which Creative Commons licence will apply to this Work are set out in our licence referred to above.

Other than as permitted in any relevant BMJ Author's Self Archiving Policies, I confirm this Work has not been accepted for publication elsewhere, is not being considered for publication elsewhere and does not duplicate material already published. I confirm all authors consent to publication of this Work and authorise the granting of this licence.

For peer review only

A FIRST LINE MANAGEMENT TEAM´S STRATEGIES FOR SUSTAINING RESILIENCE IN A SPECIALISED INTENSIVE CARE UNIT – A QUALITATIVE OBSERVATIONAL STUDY

Authors

Karl Hybinette[1,2], Karin Pukk Härenstam[1-3], Mirjam Ekstedt[1, 4]

Affiliation

[1] Department of Learning, Informatics, Management and Ethics, Karolinska Institute, Stockholm, Sweden

[2] Theme Children & Women´s Health, Karolinska University Hospital, Stockholm, Sweden

[3] Paediatric Emergency Department, Karolinska University Hospital, Stockholm, Sweden

[4] Department of Health and Caring Sciences, Linnaeus University, Kalmar, Sweden

Corresponding author

Karl Hybinette

Karolinska Institute

Department of Learning, Informatics, Management and Ethics,

Tomtebodavägen 18 A

SE 171 77 Stockholm

E-mail: karl.hybinette@ki.se

Phone: 0046704297173

For peer review only

For peer review only - http://bmjopen.bmj.com/site/about/guidelines.xhtml

ABSTRACT

Objectives

Acute care units manage high risk patients at the edge of scientifically established treatments and organisational constraints while aiming to balance reliability to standards with the needs of situational adaptation (resilience). First line managers are central in coordinating clinical care. Any systemic brittleness will be evident only in retrospect through for example care quality measures and accident statistics. This challenges us to understand what successful managerial strategies for adaptation are and how they could be improved. The managerial work of balancing reliability and adaptation is only partially understood. This study aims to explore and describe how system resilience is enhanced by naturally occurring coordination performed in situ by a management team under variable circumstances.

Design

An explorative observational study of a tertiary Neonatal Intensive Care Unit (NICU) in Sweden. One year of broad preparatory work followed by focused shadowing observations of coordination analysed through inductive-deductive content analysis from a perspective of resilience engineering.

Participants

A team of managers (i.e. clinical coordinators, head nurses, senior medical doctors).

Results

The results describe a functional relationship between the NICU´s level of manoeuvrability and a progression of adjustments in the actual situation, expressed through recurring patterns of adaptation. Managers focused on maintaining coherence in escalating problematic situations by facilitating teamwork through goalsetting, problem-solving and circumventing the technical systems' limitations.

Conclusions

For peer review only - http://bmjopen.bmj.com/site/about/guidelines.xhtml

Coordination supports a coherent goal setting by increased team collaboration and is supported by team members´ abilities to predict the behavior of each other. Our findings suggest that in design of future research or training for coordination, the focus of assessment and reflection on adaptive managerial responses may lie on situations where the system was "stretched" or "needed reorganization" and that learning should be about whether the actions were able to achieve the short-term goals while preserving the long-term goals.

Keywords

Qualitative research, Health services administration & management, Health policy, Organisation of health services, Risk management

Wordcount

4877

Strengths and limitations of this study

- The explorative ethnographic design allowed for a deeper understanding of the underspecified (hidden) work of first line managers.
- Several iterations of data collection and analysis allowed for an initially wide and later more focused data collection that opened opportunities for the researchers to follow up on specific findings.
- The inductive-deductive analysis allowed the researchers to follow and describe patterns of recurring codes within the whole dataset.
- The first author (KH) who conducted the fieldwork is an experienced neonatal intensive care nurse which may have affected the interpretations drawn in this study.
- The explorative study design was suitable for describing a complex process without measurable clinical outcomes.

For peer review only - http://bmjopen.bmj.com/site/about/guidelines.xhtml

BACKGROUND

The neonatal intensive care unit (NICU) is a specialized intensive care unit that serves as its own emergency department, intensive care unit and a pre- and postoperative ward, requiring a wide array of interrelated multi-professional teams to operate in a coordinated fashion. The many roles of the unit are coupled with the pressures of maintaining high-quality and individualized care, family support and safe care practices. Some situations are manageable by reorganizing teams internally while others require cooperation with external units.

The NICU is distributed over three hospitals in an integrated network of independent units organized under one head of department. Daily operations are overseen by the strategic operations manager to ensure collaboration for overall performance across the three units. The overarching goals as understood by the authors are individualized care, family support, high quality care and safe care practices (Figure 1).

[Insert figure 1 here]

Maintaining quality and safety is a persistent problem considering acute admissions, staff shortage and rapidly deteriorating patients with life-threatening conditions (1,2). Safety is often defined as one of many variations of ‘the absence of preventable harm to a patient during the process of health care’ (3). During the last decades of patient safety research a movement towards an inclusive view of supporting the healthcare systems´ ability to sustain required (normal) operations has emerged, the resilience engineering (RE) perspective (4). An important point made within the RE perspective is that safety can be enhanced by a combination of structure and control on one hand and adaptations on the other (5).

For peer review only - http://bmjopen.bmj.com/site/about/guidelines.xhtml

Healthcare is to be understood as a complex sociotechnical system (6). It is not only defined by the boundaries of physical locations (7). People with their skills and relationships, rules, regulations and even work place culture may impact system performance (6). Research on resilience in healthcare suggest that successful as well as unsuccessful outcomes emerge from the fluid arrangement of system components (coordination) (8). Coordination is defined as "*…the deliberate and orderly alignment or adjustment of partners' actions to achieve jointly determined goals"* (9). 'Partners' in the healthcare setting are those who share a common goal of delivering care to patients. People who perform coordinative work are a part of a "*process by which team resources, activities, and responses are organized to ensure that tasks are integrated, synchronized, and completed within established temporal constraints"* (10).

Although coordination is an integral part of everyday healthcare management we know very little about how *managers* learn the strategies they use and which of the work practices they develop that are successful (11,12).

This study aims to explore how system resilience is enhanced by naturally occurring coordination performed in situ by a management team (i.e. clinical coordinators, head nurses, senior medical doctors) under variable circumstances.

METHODS

Design and setting

This study uses an explorative ethnographic design using participatory observations and an abductive approach to capture and analyse naturally occurring coordination in situ (13). The reason for focusing on action-interaction was to capture a deep understanding of the varying conditions under which

For peer review only - http://bmjopen.bmj.com/site/about/guidelines.xhtml

108 decisions and coordination took place (14). The Standards for Reporting Qualitative Research (SRQR)
109 checklist was used to improve the transparency of research (15).

110 The study took place in a tertiary level NICU with an approximate capacity of 70 cots divided over three
111 wards located in separate hospitals. The patient mix of the three wards is dependent on local factors
112 such as the size and risk profile of adjacent delivery wards and the availability of paediatric surgical
113 capacity. Staffing for the high acuity patients is normally one nursing team per two patients (one nurse
114 and one assistant nurse). Paediatricians and neonatologists are allocated over the three wards
115 depending on availability and competence.

116 Each of the three wards is managed by a clinical coordinator, a head nurse and an operations manager
117 during daytime (Figure 1). The clinical coordinator performs tasks such as rostering, planning for patient
118 flows (admissions, discharges and transports) and does clinical work when needed. The head nurse is
119 formally responsible for the work environment and quality of care. The operations manager is a senior
120 neonatologist with an overall responsibility for the medical quality and patient flow. A strategic
121 operations manager has mandate to move patients between hospitals within the own organisation or to
122 hospitals outside the region. All managers are clinical specialists (nurses and neonatologists). Hereafter
123 we will refer to this team as the management team.

124 **Data collection**

125 Data was collected between January and February 2017. The head of the department gave permission
126 to conduct participant observations at the unit. All staff was informed about the procedures in a staff
127 meeting and they were informed that they could decline from being observed at any time according to
128 the ethics approval. The clinical coordinators (four individuals) gave written consent to being shadowed

For peer review only - http://bmjopen.bmj.com/site/about/guidelines.xhtml

after receiving written and oral information. The coordinators were all women, experienced nurses with more than 10 years of NICU experience.

Data collection was structured in iterative cycles of collection and analysis, starting with descriptive observations to get familiar with the work environment and relevant aspects of the managers' work (16). The descriptive observations focused primarily on ´places´ were the coordination of work was apparent, for example at the head nurses and secretaries open office area and at the management teams´ office at the center of the ward. The intermittent recording and analysis of field notes during initial observations yielded research questions that were in focus during the following observations.

The focused observations targeted selected situations such as rostering for the next shift, start-up meetings and handovers between shifts in addition to shadowing of coordinators. Data was collected through ad-hoc interviews with staff and managers during or after the shadowing (16). Shadowing meaning "following people, wherever they are, whatever they are doing" (17). Artefacts, including coordinators' notes on patients' medical status, occupancy- and rostering charts were copied and collected. All relevant aspects of the environment were captured in field notes during or after the observations, along with researcher's memo-writing over personal reflections and thoughts about what was happening.

Analysis

All meetings and interviews were audio recorded and transcribed verbatim. Transcriptions from tape-recorded dialogues were placed in a correct temporal order along with the field notes, so that the mix of field notes and transcription chronologically represented the full workday. Transcripts were read through several times, followed by discussions in the research group about the level of detail in the data and reflections KH had regarding the observed work shift (18). The initial inductive analysis went

For peer review only - http://bmjopen.bmj.com/site/about/guidelines.xhtml

through a two-step process (columns two and three in Table 1). First, a conversation or a situation of relevance for the study’s aim (a meaning unit) was selected and the question “What is happening here?” was directed to the data. Next, the selected conversation or situation was analysed in the context of the entire scene where it took place, with the question “Why or how is it happening here?”. The interpretation was condensed and labelled with one or several codes (Table 1). The codes and their relations were frequently discussed in the research team and sorted into tentative sub- and main categories. Field notes were included in the analysis as means to reflect on the researchers’ pre-understanding of the context. Moving back and forth between induction and deduction was a way to discover meaningful underlying patterns that made it possible to integrate concrete behaviour and deep contextual structures. Lastly, a deductive comparison of interdependencies between the main categories and sub-categories in relation to the theoretical concept of resilience was performed (14).

review only

For peer review only - http://bmjopen.bmj.com/site/about/guidelines.xhtml

Table 1 Example of the analysis in two-steps: going from *raw data* to *interpretation of incident* and to *analytical interpretation in context*, *code* and *memo*.

**Raw data**	**Interpretation of incident**	**Analytical interpretation in context**	**Code**	**Memo**
The observation begins in the flow room. The flow room is a small room with two workstations where the coordinator has her seat. There are several information sources hanging on the walls and post-it notes on the computers. The coordinator meets the observer in the flow room after having walked around the ward to check all the patient rooms.	In the physical environment, several different communication tools are gathered within a small area, i.e., tools used to summarize, remember, and disseminate important information. Information exchange occurs on paper notes stuck to computers, through software, and when the coordinator herself walks around the ward.	Information exchange is one part of coordination and can be performed through predefined channels and tools, but also more intuitively through physical meetings within the ward while the manager compares notes to what is experienced. The environment has been adapted for having several different information channels intersecting in one place.	Adapting environment – cluster tools for facilitating information exchange	The coordinator has gathered information about the current situation in the patient rooms by walking around and taking a look. She then looks at the system level, occupancy lists, and information from the other coordinators. If coordination means exercising control, real life information gathering is probably an important step.
The coordinator sits down at a computer and begins counting patients on her paper copy of the occupancy list and in TakeCare [electronic health record]. She reports the occupancy in Belport [national occupancy chart] and talks a bit about this.	Counting patients and manually entering the number into the national occupancy report is one of the first things the coordinator does in the morning.	The coordinator gets an idea of the status and distributes it to the rest of the country as information. This is proactive management, as future coordination may become easier if reference can be made to Belport, or if you know that the other units in the country your information.	Information handling	Reporting your status is a way of exercising control by impacting others perception regarding the degrees of freedom at your end.

PATIENT AND PUBLIC INVOLVMENT

Public and patient involvement was not applicable in this research.

For peer review only - http://bmjopen.bmj.com/site/about/guidelines.xhtml

165 **RESULTS**

166 The analysis resulted in six subcategories and two unifying main categories (table 2).

167 The ´how´ and ´when´ of coordination is presented in Table 2 that illustrate the functional relationship
168 between the two main categories *adjustments to meet the actual situation* and *maneuverability* with
169 their respective sub-categories. The sub-categories *Supporting system cohesion* and *Extending system*
170 *boundaries* describe the joint work of managers and clinical staff. *Adapting the structure and roles of the*
171 *coordinating management team* and *Shifting between information sources for better sensemaking*
172 describe the management team's internal work.

173 Extracts from the field notes and conversations are presented to clarify the findings. More
174 comprehensive material is provided in Appendix 1.

For peer review only - http://bmjopen.bmj.com/site/about/guidelines.xhtml

Table 2 The main category on the y-axis are *adjustments to meet the actual situation* and on the x-axis; *maneuverability.* Four sub-categories on the y-axis describe strategies (i.e. the work that managers do); and the three sub-categories on the x-axis describe a progression of a perceived level of manoeuvrability based on the expressed availability of *degrees of freedom* with illustrative quotes.

		**Manoeuvrability**		
		**Losing control – reorganisation**	**Stretching the system to work outside ordinary conditions**	**Everyday work under ordinary conditions**
**Adjustments to meet the actual situation**		”So [the other hospital] have 20 babies now, seven acute. They have opened a temporary room but have no staff for it. We have no transport team [to use as staff] because they are on their way to [another city] […] I still won't send babies to other counties, which we can't because we have no transport team available […] either we find some staff here that can go there or we have to order two on overtime. How do we usually do?” (Operations manager).	“We have put those two [patients] together in 9:1 to get an emergency cot in 9:2 for the twins [9 refers to a room divided in two sections equipped for one patient per section 9:1 and 9:2], I told the father that they may have to move out. But then we will be in the situation where they maybe… They may need… That father is very new. So, I think that in that case they will have to be two [staff] in there too.” (Clinical coordinator). [Q1 – appendix 1]	”Oh, so we are only at eight intensive care babies and three in family rooms. So, it's a pretty good situation. And we have nothing acute in the delivery- or antenatal wards. I just checked.” (Operations manager).
	**Supporting system cohesion**	• Delay work and evaluate the situation<br>• Isolate problems and focus on re-creating manoeuvrability.<br>• Exploit possibilities of extraordinary individual achievements (trade-off individual resilience for system control).	• Goalsetting towards protecting manoeuvrability.<br>• Goalsetting for promoting basic safe care practices at the clinical level through minimal staff allocation and skill mix.<br>• Sacrificing continuity in patient assignments for saving lives.	• Goalsetting towards family-centred care<br>• Goalsetting of individualised care.<br>• Managing optimal staff allocation for maintaining professional development and education.<br>• Controlling occupancy and redundant capacity through predefined strategies.<br>• Monitoring state of the ward at the clinical level by regular walkarounds in the clinical work environment.
	**Extending system boundaries**	• Identifying novel use of any existing external resources (i.e. use of paediatric emergency transport team and other wards).<br>• Shedding managerial tasks for participating in clinical emergency work (trade-off management for clinical work).	• Managing occupancy trade-offs between facilities and staffing (higher occupancy in fewer rooms lowers staff requirements).<br>• Utilising individual managers social networks within predefined limits for proactive problem-solving.	• System working within normal boundaries.
	**Adapting the structure and roles of the coordinating management team**	• Make *loss of control* explicit in the management team.<br>• Moving from understanding the situation to making rapid decisions close to the clinical level.	• Relaying information on the patients' clinical situation to mid- and upper-level managers (information priority bottom-up).	• Relaying high level plans to clinical level workers through regular briefings (information priority top-down).<br>• Participate in regular medical discussions on patients' status´.
	**Shifting between information sources for better sensemaking**	• Dropping computerised aides, rostering systems and staffing charts for handwritten notes and memos.<br>• Using face-to-face communication with people in close vicinity (shedding electronic communication).	• Seeking ad-hoc meetings within the management team for calibrating information of the situation and possible workarounds.<br>• Verbally explaining situations to other managers.	• Regular use of computerised systems and handwritten notes.

Manoeuvrability

For peer review only - http://bmjopen.bmj.com/site/about/guidelines.xhtml

Managers face situations where they must balance a limited number of staff and demands for high occupancy with the task of supporting clinical teams in their care of patients. Unpredictable factors such as acute admissions, staff availability and the medical progression of patients put managers in situations where they might quickly have to re-adjust and re-plan, often ad-hoc with scarce information of the overall situation. Part of the observed dialogue relate to the management team's efforts to identify alternative ways forward. See quotes at the head of Table 2. Further examples of the choices made under each sub-category of manoeuvrability is presented in table 3.

In the quote below, the head nurse acknowledges that the inflow of emergency patients is sometimes impossible to avoid.

"The patient flow, do you participate in that?" (Observer). "No, no more than that I can say ´stop´, because I don't have the staffing." (Head nurse). "Ok, so you can say that too?" (Observer). "Mm, I can say that I have five or six teams. But…" (Head nurse). "But you cannot say stop today." (Coordinator). "No, but it is like that. Even if we say stop the babies are being born. And we have to take care of them." (Head nurse).

Table 3 Examples of ´situations´ and ´choices made´ sorted under each sub-category of manoeuvrability.

Losing control – reorganisation	Stretching the system to work outside ordinary conditions	Everyday work under ordinary conditions
**Situation with several problematic trade-offs.**<br>The coordinator approaches the head nurse when she cannot find a suitable cot for a new patient (Field-notes).<br>"We have another problem." (Coordinator). "Right, what?" (Head nurse). "This new week 28 [patient], I cannot put in room 10 [a room with four cots, one is available], the other baby in there is terminal and about to die." (Coordinator). "But if we place him in room 16 or 15?" (Head nurse). "No, I can't put it in 15 because I have two cots in there for the twins and they must be together [also incoming patients]." (Coordinator). […] "We cannot have the twins remaining at the stabilisation unit at delivery, there might be another patient coming in." (Coordinator). "Okay, because I thought it	**Exploring options to avoid patient transfers because of staff shortage.**<br>"NN [strategic operations manager] think that we can conserve personnel by moving ´K´ [a patient] to ´Ts´ room [another patients]." (Head nurse). "Okay." (Observer). "But I am not so sure we conserve personnel by that; and the family have already moved several times, you know that they are the ones that was supposed to be moved to [another hospital]." (Coordinator). "Okay" (Observer). "It [the patient] started to have breathing problems and came back. And now after moving back here they will have to move again ´sigh´." (Coordinator).	**The deliberate adaptation of an administrative routine.**<br>"I have talked to NN [a nurse currently placed at the emergency stabilisation unit in the delivery ward] because there is a week 35 baby there that the doctor did not want to take it up to the ward during the nightshift." (Coordinator). "No?" (Observer). "Yes, then he has of course been there a little longer that his allowed six hours." (Coordinator).

For peer review only

For peer review only - http://bmjopen.bmj.com/site/about/guidelines.xhtml

would be a better idea to move the twins directly from delivery [to another hospital] if they need to move anyway at some point." (Head nurse). "Mm, but where should they move. All hospitals are full [in the county]. We would have to move them out of county." (Coordinator). "Yes, we will never be allowed to do that." (Head nurse).		

Everyday work under ordinary conditions

The management team have planned meetings; they sweep the ward to collect information on the state of things and relay high level plans to workers.

The coordinator begins her shift by conducting a walkaround of the ward, saying hello to the nurses and doctors as she is asking if everything is all right. After the walkaround she sits down to begin exploring her staff roster and patient occupancy charts (Field-notes).

Under ordinary conditions there is a minimal observable need for managers to manually adapt information they extract from technical systems, regarding for example occupancy, patient acuity levels and staffing. Managers describe how their experience of the technical systems' limitations are learned on the job and how workarounds are taught between individuals.

The coordinator makes a note of something on her occupancy chart (Field-notes). "What did you write on that chart?" (Observer). "CT 11:15 [Computer Tomography at 11:15]." (Coordinator). "And it is used for that kind of information?" (Observer). "I print one of these charts because there is more space to write on. I think… I think many… I think NN and NN [two other coordinators] use this too. And NN [another coordinator] use it because I taught her. And I use it because NN [a coordinator who quit earlier] taught me." (Coordinator). "Okay, that is nice. But is it the same information you all use it for?" (Observer). "Well, I don't know?" (Coordinator). "Not important?" (Observer). "Well, I don't think so, I use it for… those that are planned for other hospitals, delivery ward, antenatal… Oh! I forgot to write that today [makes another note on the chart] and stuff that I need to do." (Coordinator).

For peer review only - http://bmjopen.bmj.com/site/about/guidelines.xhtml

212 ***Stretching the system to work outside ordinary conditions***

213 The following situation is a typical example of how the management team must re-organize and adapt 214 to stretched conditions. The ad-hoc meeting in the example illustrates that strategic goals of family 215 centred care and optimal staff allocation are traded for immediate medical priorities, basic safe care 216 practises and protection of the wider system. Other patients on the ward are cared for by their 217 respective teams, avoiding exposure of this emerging crisis because it is handled by the paediatric 218 emergency transport team.

219 *The plan for this day is to admit one intermediary level patient that was born during the night and is* 220 *waiting for a cot at the NICU. One patient has been diagnosed with a multi-resistant bacterium and will* 221 *need increased hygiene standards (Field- notes) […] "We are plenty of people today, which is nice. But* 222 *when NN [strategic operations manager] asked if we had a lot of capacity, I had to say no." (Clinical* 223 *coordinator). […] "...one important thing. This baby that needed eye surgery is now acute and will arrive* 224 *soon. They will land directly in the operating room and the transport team will take care of the baby until* 225 *it can go back home to its own hospital. (Operations manager).* [Q2 – Appendix 1]

226 The plan for postoperative care after acute eye-surgery was for the patient to be assigned a cot and a 227 nursing team on the ward. The situation was managed by using the transport team to temporarily care 228 for the baby inside the operating room until it was stable enough for transport to another hospital. This 229 decision had possible implications for the whole management team. The clinical coordinator wanted to 230 know about the utilisation of staff and facilities. The head nurse about workplace safety, quality of care 231 and economy. The operations manager had responsibility for the medical quality and the strategic 232 operations manager for the possibility of helping one of the other wards with staffing.

233 ***Losing control – reorganisation***

For peer review only - http://bmjopen.bmj.com/site/about/guidelines.xhtml

The category of ´losing control´ was characterised by managers shedding managerial tasks for participating in clinical emergency work, isolating problems and focusing on re-creating manoeuvrability. The focus was to protect clinical teams from being exposed to the rapidly shifting plans and priorities at the managerial level. Strategies for regaining control can be to sacrifice the goal of continuity by transferring at-risk mothers to other hospitals (deferral), or to temporarily transfer additional weight of medical care to neighbouring sub-systems such as the paediatric emergency transport team or the managers themselves.

The following situation illustrates a reorganisation at the verge of losing control. The coordinator tried to reserve an empty cot for possible additional acute emergency admissions.

"This is not good, its full [the ward]. We have no space when this eye baby arrives." (Clinical coordinator). The clinical coordinator walks to the room where a nurse oversees the twins that were supposed to be transported out the next day but are now showing symptoms of infection. (Field- notes). "Do you think these twins could be together in a twin cot?" (Clinical coordinator) […] "Well, I don´t know. This one is just on the margin of managing without incubator." (Nurse) […] "The problem is that I don't have staff to open another room." (Clinical coordinator). [Q3 – Appendix 1]

The manager handled the situation by putting twins together in one cot, thereby utilising one nurse to care for three babies which is more than the goal of two babies per nurse. This manoeuvre created an opportunity to temporarily handle five patients (with one empty emergency cot) in a room with staffing for four. The nurse expressed concern for her patient but remained focused on finding a solution.

When the managers started shedding managerial tasks for bedside operative work, they risked losing the ability to meet other managers and keep up to date with the ward. Management decisions in these situations were then based on a narrower understanding of the bigger picture. Computer aides were less used (or not at all) because of their inability to present rapidly changing borderline conditions.

For peer review only - http://bmjopen.bmj.com/site/about/guidelines.xhtml

Adjustments to meet the actual situation

It was observed how the mandate for making decisions was distributed across the management team and clinical nursing teams (speech bubbles in figure 2). The clinical coordinator moved freely across the unit and made independent decisions regarding resource allocation, rostering and whether to shed her own managerial work to help the clinical teams. The clinical coordinator also provided the other managers with information when they needed to adjust their plans.

[Insert figure 2 here]

Supporting system cohesion

Members of the management team were observed to often communicate through ad-hoc meetings where verbal information was compared to patient rosters, patient conditions, staffing and workload indicators. They worked close to the clinical context and discussed the current goals' attainment for individual clinical teams and what the current trade-offs were. The managers compared computer- and paper-based notes, as well as information provided at start-up meetings to update themselves of the status of the ward and where the situation was heading (umbrella perspective in Figure 2).

The coordinator is back in room nine to alleviate the staff for lunch. She checks in on the baby that has been in acute surgery for intestinal obstruction. There are lots of beeping sounds, but no one seems alarmed. The head nurse enters the room and seeks the coordinators attention, but initially fails as the coordinator is tending to a patient. Eventually the coordinator looks up (Field-notes). "Didn't they [the surgeons] say already this morning that this patient was up for re-operation?" (Coordinator). "After lunch apparently. Then what do we do with NN [another patient]?" (Head nurse).

In the example above the managers discussed how two patients' trajectories were affected by the surgeons rescheduling. The management team negotiated situations that needed simultaneous

For peer review only - http://bmjopen.bmj.com/site/about/guidelines.xhtml

attention, like prioritizing readiness and clinical capacity in some parts of the ward while maintaining family centered care and staff education in others (Illustrated as the three clinical teams in Figure 2).

Extending system boundaries

The need for extending the system's boundaries emerged as the pressure of prioritizing decisions increased. The management team made use of other units' facilities or staff, like delaying patients in the operating theatre or letting a transport team care for the patient for some time before handing it over to the regular staff. The management team utilised auxiliary staff and overlapping competencies of different professional groups. Sometimes the managers themselves doubled as clinical staff within their vocation.

"It is already a little tight if it arrives [the surgery baby], right now we have low workload in room nine and ten with four patients and the other goes back to [another hospital out in another region] at five [in the afternoon]. We hope. But it is also, I mean, if there is an acute admission and he... I do not think NN [a nurse] can have four patients by himself out there. Since the father needs quite a lot of help." (Coordinator). "They are really good patients those babies" (Operations manager). "What about the midwives then? [that belongs to the adjacent delivery ward]" (Coordinator). "There are four of them [turns to face the operations manager]?" (Head nurse). "Yesterday someone said that you can have four patients by yourself." (Operations manager). "Not by yourself" (Coordinator). "With an assistant nurse." (Head nurse). "Yes with an assistant yes, that is okay but." (Coordinator). "So there are three patients left?" (Head nurse). "And then we have put two in the same cot at 9:1 [bed one in room nine]." (Head nurse 2). "Okay." (Head nurse).

Adapting the structure and roles of the coordinating management team

For peer review only - http://bmjopen.bmj.com/site/about/guidelines.xhtml

The management team fluently adapted its own structure in situations where there were not enough resources to manage within everyday routines, when there was urgency or when some of the management team members were not available with their specific expertise and mandate. This structural adaptation was observed when individuals in the management team shifted from relaying plans from top-down to working with patients and gathering information from bottom-up.

"In room nine is that week 22 baby that came in yesterday. They are intubating now so they use a lot of people. There were no head nurses here at seven, so I decided myself that NN [nurse] got to be alone at the stabilisation room [at the delivery ward]. The doctors there have to work a little harder now." (Clinical coordinator).

Shifting between information sources for better sensemaking

As the situation on the ward became more complex the management team increased their reliance-on handwritten notes rather than the standard computer-generated lists for staffing and patient acuity information.

The coordinator used handwritten notes as memory aides in team discussions. The notes were mainly short markings, phrases, or single words in the margins like "discharge planned" or "need antibiotics". The limitations of computer-generated patient rosters to convey this type of information on real world complexities were expressed by members of the management team.

The national occupancy chart for example was only able to classify patients as high or low acuity without regards of other factors. When there was a need to work outside the binary world of two patient groups, the team stopped using this computer-generated aide and instead relied on their own domain knowledge, personal network and the stability of the management teams' understanding of the bigger picture.

For peer review only - http://bmjopen.bmj.com/site/about/guidelines.xhtml

“Well and this is actually look correct [number of beds in the national occupancy chart] 14 in total. But with the stabilisation beds. That makes it 12 here plus two there… But it is also a little [inaccurate]. Because then you calculate [all of them as intensive care beds]. The stabilisation beds are supposed to be low acuity”. (Clinical coordinator)

The following quote illustrates what happens when the computerised information indicated normal occupancy when the off-going night nurse reported understaffing for the same shift.

“How did this happen?” (Clinical coordinator).

”Well, because NN [one of the nurses] who is work-training after sick leave was included in the staffing. The parenthesis was probably put there later [points at the handwritten parenthesis in the rostering folder, indicating that NN should not be included in the staffing]”. (Night nurse)

For peer review

DISCUSSION

The management team in this study exhibited a range of mindful adaptations for sustaining the units´ capacity for expressing resilience. Such as sacrificing low level goals based on up-to date information and continuous assessments of what would be minimally intrusive for the overall performance of the system (figure 2). Our findings elucidate a link between adjustments to meet the actual situation and the available manoeuvrability of the system.

Supporting coherence

The management team aimed to balance the demands and capacity of multiple teams that operate in separate rooms while tending to patients with a wide variety of problems and acuity. For practical reasons the teams could not always meet to communicate with each other. A defining characteristic of a

For peer review only - http://bmjopen.bmj.com/site/about/guidelines.xhtml

multiteam system is the ability of component teams to modify individual goal hierarchies while sharing a common distal goal or set of goals (12). During the observations the management team and the clinical staff consequently agreed on making provision of acute care to rapidly deteriorating patients a top priority, allowing us to identify it as a core mission (i.e. purpose of the system) (19). Other priorities were more likely to be put on hold and resumed later or to be permanently dropped.

When the care teams were unable to communicate, maintaining coherence was an important factor for the managers´ maintenance of an umbrella perspective i.e. what they needed in order to understand how the bigger picture of their interventions fit together (20). At this point, we can say two things about coherence, with implications for the training of managers as adaptive teams (21). First, that coordination supported a coherent goal setting with increased team collaboration and second, that it was enhanced by team members' ability to predict the most likely priorities of each other.

Reorganising to support manoeuvrability

Everyday work of the management team was characterised by seamlessly and actively organising and reorganising. Our observations illustrate how the management team made use of early investments in for example staff´s expertise, deep domain knowledge and the workplace culture to maintain a unit wide focus on the core mission (22). Allowing the care teams to adapt their goals individually exemplifies that being resilient is to be part of a process of identifying conflicting goals in a complex, intractable environment using *"numerous indicators in a proactive fashion to probe a system's adaptive capacity before system-wide collapse results in disaster"* (21). A realisation from studying the management team was the shapelessness of the organisation. We could not observe a formal agenda for how and why the management team was supposed to prioritize in terms of goal achievement below the core mission. Our study suggests that it is up to the management team to support the system by using experience, professional ethos and domain knowledge to negotiate the way forward in a manner

For peer review only - http://bmjopen.bmj.com/site/about/guidelines.xhtml

that resembles the fifth principle of managing the unexpected, as described by Weick and Sutcliffe (23). Specifically, because the flexible decision structures enabled resilient performance when expertise and experience outranked formal hierarchical positions.

Important for the application of resilience in everyday clinical work was the link between adaptation and outcome (successful or unsuccessful) as described in for example the CARE model for researching resilience in healthcare (24). An adaptation is a deviation from work as planned, and it is not always clear beforehand whether the outcome of an adaptation constitutes success or failure in terms of quality and safety. Our study describes managers' adaptive responses to the conflicting demands of acute patient care on one hand and long-term strategic demands on the other (measured as for example respirator days, patient throughput and hospital acquired bloodstream infections).

Balancing between long- and short-term goals

Resilience depended on the use of earlier investments in 'potential opportunities for action' previously described as degrees of freedom. However, low-level goal-sacrifices do represent a loss of potential future degrees of freedom if it is overexploited. In the context of the NICU, families are less prepared for discharge if they are not trained, staff might receive less time learning from experienced colleagues if they do not work together and formal routines might erode if they are not employed.

The balancing act between seemingly irreconcilable goals makes it impossible to decide in retrospect whether coordination was good or bad for the total outcome of the system. Each decision to suspend or sacrifice a low-level goal has implications for the organisation's future capacity for expressing resilience. All teams worked towards the core mission of providing acute care. However, the maintenance of long-term investments was achieved by managing a diversity of low-level goal-sacrifices between many clinical teams (i.e. sacrificing patient education in one and staff education in another). Our findings

For peer review only - http://bmjopen.bmj.com/site/about/guidelines.xhtml

suggest that in design of future research or training for coordination, the focus of assessment and reflection should be on adaptive managerial responses in situations where the system is ‘stretched’ or ‘in need of reorganization’ (table 2). Furthermore, learning should be about whether the actions lead to achievement of short-term goals while preserving the long-term goals.

Limitations

This was a single centre study of a specialised unit with a specific patient clientele that cannot be cared for by any other type of unit available. It was expected that the unit´s high tempo and specialisation would promote a particularly observable coordinative work with the risk that it might introduce the argument of limited transferability to other areas of healthcare. We believe however that the networked structure of three wards is not unique. The specific unit for study enabled us to capture and understand the subtleties of everyday work of first line managers. Further studies are needed to investigate how much of this work may be specific to organizations of both similar and contrasting types.

Using a qualitative cross-sectional design, this study does not allow us to define successful or unsuccessful outcomes. Resilience is described based on the actions taken and further studies are required to operationalise and test our results.

The first author (KH) who conducted the fieldwork is an experienced neonatal intensive care nurse with experience from the studied NICU. The familiarity with the specific type of work may have affected the interpretations drawn in this study.

Trustworthiness during data analysis was addressed by regular peer-check and in seminars with the wider research group and member check. The final analysis was individually validated with the coordinators (25). The iterative process of data collection and analysis was intended to ensure that the

For peer review only - http://bmjopen.bmj.com/site/about/guidelines.xhtml

analysis included more than one researcher's interpretation. Transferability was addressed by leaving an audit trail of extracts from the data in the report so that readers from other fields can evaluate if the results are transferable to their respective contexts (26). Table 1 of the methodological section provides a trail of how interpretations of data were made.

The use of voice recordings of meetings and conversations was limited to situations where verbal consent could be obtained from all participants, unless explicitly asked not to by any of the participants (27). In larger groups, where participants attended only partially this was not a feasible option and handwritten notes were taken, video recordings were not possible because of difficulties with assuring patient anonymity in the clinical context.

CONCLUSIONS

We are only beginning to understand managers' strategies for prioritising and acting on the variability of degrees of freedom. Our findings suggest that managers at the clinical level, while being central to the system's capacity for expressing resilience, do not have an explicit model or training for how they approach their work. Furthermore, managers lack the aid of tailored decision support systems. This could depend on well described challenges for design of such interventions (28). It is important that healthcare policy and organisational redesign initiated at higher levels are well calibrated with the nature of managerial work on the clinical level before interventions can be developed. Our recommendations for future research and policy making is to prioritise definition and operationalisation of successful coordination in a wide variety of contexts.

For peer review only - http://bmjopen.bmj.com/site/about/guidelines.xhtml

LIST OF ABBREVIATIONS

RE: Resilience Engineering;

NICU: Neonatal Intensive Care Unit

DECLARATIONS

Ethics approval

This study was approved by the regional ethical review board of Stockholm (2016/1832-32).

Consent for publication

Not applicable.

Availability of data and material

Given that the data is in Swedish and the phrasing of the consent obtained from participants, complete raw data are not available for public sharing. Partial datasets used and/or analysed during the current study are available from the corresponding author on reasonable request. A selection of translated quotes is supplied in the results and methods sections and in appendix 1.

Competing interests

The authors declare that they have no competing interests.

Funding

The authors received no specific funding for this work.

Author Contributions

For peer review only

For peer review only - http://bmjopen.bmj.com/site/about/guidelines.xhtml

The study was initiated by KH. The study design was developed in collaboration within the research team. KH performed the data collection. The analysis and interpretation of data was conducted in close collaboration between KH, ME and KPH. KH wrote the first draft of the paper. All authors contributed with writing, critical revisions and approval of the final version.

Acknowledgement

Thank to Professor Eric Hollnagel that contributed with theoretical expertise on the interpretation of data; Professor Richard Cook for valuable discussions about system safety and methodology in the early phase of the study. Thanks also to the participating Neonatal unit.

References

1. Samra HA, McGrath JM, Rollins W. Patient safety in the NICU: A comprehensive review. J Perinat Neonatal Nurs. 2011;25(2):123–32.

2. Bondurant PG, Nielsen-farrell J, Armstrong L. The Journey to High Reliability in the NICU. J perinaltal Nurs. 2015;29(2):170–8.

3. Kim L, Lyder CH, Mcneese-Smith D, Leach LS, Needleman J. Defining attributes of patient safety through a concept analysis. J Adv Nurs. 2015;71(11):2490–503.

4. Wears RL, Hollnagel E, Braithwaite J. Resilient Health Care, Volume 2 : The resilience of everyday clinical work. Farnham: Ashgate Publishing Limited; 2015. 328 p.

5. Eurocontrol. From Safety-I to Safety-II: A White Paper. Netw Manag. 2013;1–32.

6. Braithwaite J, Churruca K, Ellis LA, Long J, Clay-williams R, Damen N, et al. Complexity Science in Healthcare – Aspirations, Approaches, Applications and Accomplishments: A White Paper. Australian Institute of Health Innovation, Macquarie University. Sydney; 2017.

For peer review only - http://bmjopen.bmj.com/site/about/guidelines.xhtml

7. Cilliers P. Boundaries, Hierarchies and Networks in Complex Systems. Int J Innov Manag [Internet]. 2001;05(02):135–47. Available from: http://www.worldscientific.com/doi/abs/10.1142/S1363919601000312

8. Cook RI. Taking Things in One's Stride: Cognitive Features of Two Resilient Performances. In: Hollnagel E, Woods DD, Leveson N, editors. Resilience Engineering: Concepts and Precepts [Internet]. Burlington: Ashgate; 2006. p. 205–21. Available from: http://qualitysafety.bmj.com/lookup/doi/10.1136/qshc.2006.018390

9. Gulati R, Wohlgezogen F, Zhelyazkov P. The Two Facets of Collaboration: Cooperation and Coordination in Strategic Alliances. Acad Manag Ann [Internet]. 2012;6(1):531–83. Available from: http://search.ebscohost.com/login.aspx?direct=true&db=bth&AN=77495192&site=ehost-live&scope=site

10. Cannon-Bowers J, Tannenbaum S, Salas E, Volpe C. Defining team competencies: Implications for training requirements and strategies. In: Guzzo R, Salas E, Associates, editors. Team effectiveness and decision making in organizations. San Francisco, CA: Jossey-Bass; 1995. p. 333–80.

11. Cook R, Woods D. The messy details: Insights from technical work studies in healthcare. In: Proceedings of the human factors and ergonomics society 47th annual meeting. 2003. p. 2002–3.

12. Mathieu J, Marks MA. Multiteam Systems. In: Anderson N, Ones D, Sinangil HK VC, editor. Handbook of Industrial, Work & Organizational Psychology. London, England: SAGE Publications; 2001. p. 289–313.

13. Silverman D. Interpreting Qualitative Data: Methods for Analysing Talk, Text and Interaction. 2 nd. London, England: SAGE Publications; 2001. 325 p.

14. Dubois A, Gadde LE. Systematic combining: An abductive approach to case research. J Bus Res.

For peer review only

For peer review only - http://bmjopen.bmj.com/site/about/guidelines.xhtml

2002;55(7):553–60.

15. O'Brien BC, Harris IB, Beckman TJ, Reed DA, Cook DA. Standards for reporting qualitative research: A synthesis of recommendations. Acad Med. 2014;89(9):1245–51.

16. Spradley JP. Participant observation. New York: Holt, Rinehart and Winston; 1980. 195 p.

17. Arman R, Vie OE, Åsvoll H. Refining shadowing methods for studying managerial work. In: The Work of ManagersTowards a Practice Theory of Management [Internet]. Oxford University Press; 2012. p. 301–17. Available from: http://www.oxfordscholarship.com/view/10.1093/acprof:oso/9780199639724.001.0001/acprof-9780199639724-chapter-16

18. Strauss A, Corbin J. Basics Of Qualitative Research: Techniques And Procedures For Developing Grounded Theory. 4 th ed. London: SAGE Publications; 1998. 456 p.

19. Vicente KJ. Cognitive work analysis : toward safe, productive, and healthy computer-based work. London, England: Lawrence Erlbaum Associates; 1999. xix, 392 p.

20. Goldenhar LM, Brady PW, Sutcliffe KM, Muething SE. Huddling for high reliability and situation awareness. BMJ Qual Saf. 2013;22(11):899–906.

21. Gorman JC, Cooke NJ, Amazeen PG. Training adaptive teams. Hum Factors. 2010;52(2):295–307.

22. Ekstedt M, Cook RI. The stockholm blizzard of 2012. In: Resilient Health Care, Volume 2: The Resilience of Everyday Clinical Work. 2017. p. 59–74.

23. Weick KE, Sutcliffe KM. Managing the expected: Sustained performance in a complex world. 3rd ed. Wiley; 2015. 224 p.

24. Anderson JE, Ross AJ, Jaye P. Modelling Resilience and Researching the Gap between Work-as-

For peer review only

For peer review only - http://bmjopen.bmj.com/site/about/guidelines.xhtml

Imagined and Work-as-Done. In: J. Braithwaite, R. L. Wears RL and EH, editor. Resilient Health Care, Volume 3, Reconciling Work-as-Imagined and Work-as-Done. Farnham: Ashgate; 2017. p. 133–41.

25. Lincoln YS. Emerging Criteria for Quality in Qualitative and Interpretive Research. Qual Inq [Internet]. 1995 Sep 29;1(3):275–89. Available from: http://methods.sagepub.com/book/the-qualitative-inquiry-reader/n19.xml

26. Elo S, Kääriäinen M, Kanste O, Pölkki T, Utriainen K, Kyngäs H. Qualitative Content Analysis. SAGE Open. 2014;4(1):215824401452263.

27. Johnson B. Ethical issues in shadowing research. Qual Res Organ Manag An Int J [Internet]. 2014;9(1):21–40. Available from: http://www.emeraldinsight.com/doi/10.1108/QROM-09-2012-1099

28. Praetorius G, Hollnagel E, Dahlman J. Modelling Vessel Traffic Service to understand resilience in everyday operations. Reliab Eng Syst Saf. 2015;141:10–21.

Figure legends

Fig. 1 The studied NICU in a tertiary level hospital with surgical capacity. Managers are visually located to illustrate their vicinity to the clinical care teams. Overarching goals are presented at the base of the figure with examples of how they are expressed in daily work. A selection of peripheral wards and units are illustrated in the surrounding area. The arrows are double headed to symbolise a two-way relationship of demands and possibilities for negotiation of for example patient transfers.

Fig. 2 Illustration of the work of balancing goal-settings for system wide coherence, maintaining the umbrella perspective and streamlining communication to meet rapidly changing demands with the shadowed clinical coordinator at the centre. The width of double headed arrows visualises an estimation of the most frequently observed communication for the respective manager.

For peer review only

For peer review only

For peer review only - http://bmjopen.bmj.com/site/about/guidelines.xhtml

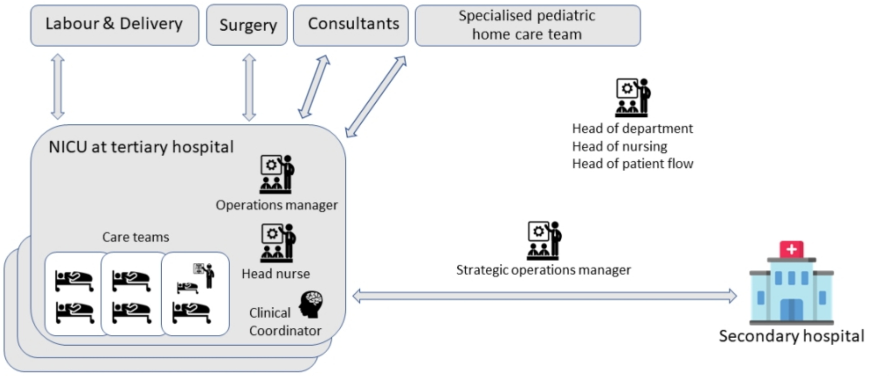

**Individualised care**	• Individualised care plans<br>• Individually assigned nurse in charge of planning
**Family support**	• Family support is a mandatory part of individual care plans<br>• Follow-up in quality assurance program (All families are offered to participate in evaluation survey)
**High quality specialised care**	• Participation in National Perinatal Quality Register<br>• Continuous nurse led quality assurance programs in areas such as nutrition, pain management and hygiene
**Safe care practises**	• Patient safety organisation including patient safety briefings, internal education/training and newsletter<br>• Scorecard with key performance indicators: Mortality, morbidity after severe illness and iatrogenic conditions such as hypothermia, intraventricular brain haemorrhage and hospital acquired infection

Fig. 1 The studied NICU in a tertiary level hospital with surgical capacity. Managers are visually located to illustrate their vicinity to the clinical care teams. Overarching goals are presented at the base of the figure with examples of how they are expressed in daily work. A selection of peripheral wards and units are illustrated in the surrounding area. The arrows are double headed to symbolise a two-way relationship of demands and possibilities for negotiation of for example patient transfers.

108x60mm (300 x 300 DPI)

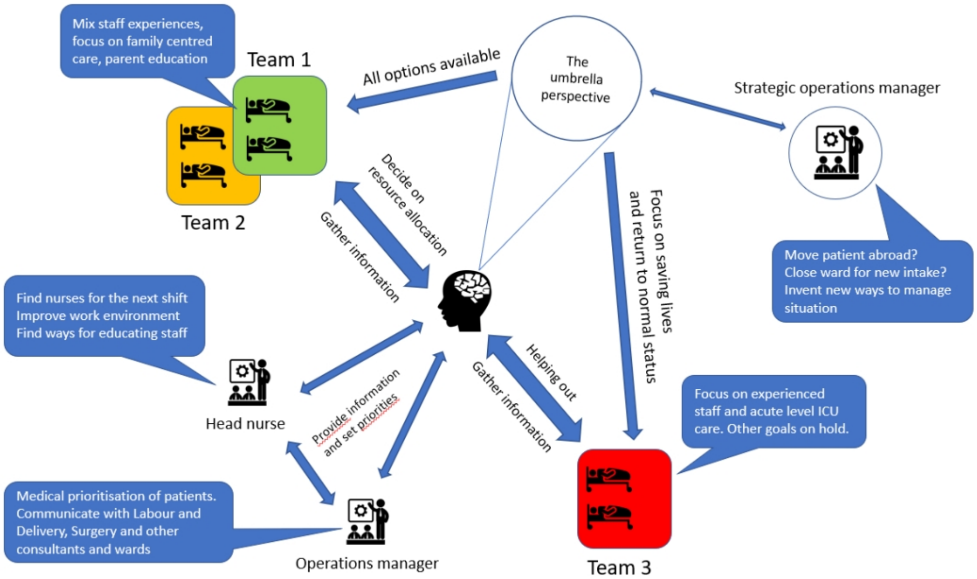

Fig. 2 Illustration of the work of balancing goal-settings for system wide coherence, maintaining the umbrella perspective and streamlining communication to meet rapidly changing demands with the shadowed clinical coordinator at the centre. The width of double headed arrows visualises an estimation of the most frequently observed communication for the respective manager.

102x61mm (300 x 300 DPI)

Appendix 1

Q1

(Clinical coordinator) We have put those two [patients] together in 9:1 to get an emergency cot in 9:2 for the twins [9 refers to a room divided in two sections equipped for one patient per section 9:1 and 9:2], I told the father that they may have to move out. But then we'll be in the situation where they maybe… They may need… That father is very new. So, I think that in that case they'll have to be two [staff] out there too.
(Operations manager) The [NN] twins?
(Head nurse) Right. Now there are two kids there, so one person can take care of them. But if you add those two [twins] then there'll be four babies, so then you need another person out there [on the ward] and there'll only be two staff left in there [room 9].
(Operations manager) Mm, it's tricky.
(Head nurse) Oh... so there are three of them who can help each other and… in [room] 7 and 8?
(Operations manager) Okay… I understand that to mean that we have a bit of leeway here if we need it in an emergency... but nothing that we can give to anyone else. Okay. [previously wanted to lend a nurse to another ward in need]
(Head nurse) Yeah, that's too bad. We'd love to be able to.
(Operations manager) Take over week 36 twins who need it, say. And then send them back, that's no fun.
(Clinical coordinator) And then we'd have to open like [rooms] 13 and 14.

Q2

The coordinator calls for an ad-hoc on-site face-to-face discussion with the strategic operations manager. This happens when the CMT experience that basic safe care practices at the clinical level consume every opportunity for maintaining overarching quality goals, the unit stop lending help to others and start sacrificing continuity of care for individual patients. The plan for today is to admit one intermediary level patient that were born during the night and is waiting for a room at the NICU. One patient has been diagnosed with a multi-resistant bacterium and will need increased hygiene standards. One baby currently in another hospital are being assessed by a surgeon for the need of possible transport and surgery, one will possibly arrive by helicopter for eye surgery today. Three babies are planned for being transported back to their respective local hospitals, one by helicopter today and twins tomorrow (Observers notes after the coordinating management team's morning huddle).

- *(Clinical coordinator) We are plenty of people today, that is nice. But when NN [strategic operations manager] asked if we had a lot of capacity, I had to say no.*

The clinical coordinator checks the antenatal list of at-risk mothers who are under observation.

- *(Clinical coordinator) Oh, there is a lot here, induction, ich... a lot of bleedings. There is a lot waiting... but you don't know when... It could be calm... I will not read all their charts.*
- *(Operations manager) ...one important thing. This baby that needed eye surgery is now acute and will arrive soon. They will land directly in the operating room and the transport team will take care of the baby until it can go back home its own hospital.*
- *(Strategic operations manager) Can we lend out some staff to [a neonatal ward in a neighbouring hospital].*
- *(Head nurse) I can check, we have plenty of staff, but they are all in use.*

For peer review only

- *(Strategic operations manager) I ask because they are not doing so well [the other unit]. Twins are being born and they are asking for allowance and resources to move them to another region.*

Q3

- *(Clinical coordinator) This is not good, its full [the ward]. We have no space when this eye baby arrives.*

The clinical coordinator walks to the room where a nurse oversees the twins that were supposed to be transported out the next day but are now showing symptoms of infection.

- *(Clinical coordinator) Lets´ see when [Strategic operations manager] gets back, maybe we can send someone else.*
- *(Nurse) But they went to fetch the week 31 from delivery to there [points to an empty cot].*
- *(Clinical coordinator) Yes, and the eye baby is coming [points toward the 2nd empty place for a cot. Then its full here. Do you think these twins could be together in a twin cot?*
- *(Nurse) Well, I don´t know. This one is just on the margin of managing without incubator, and that one is getting treatment for bilirubin [treatment includes being exposed to light from a special lamp].*
- *(Clinical coordinator) The problem is that I don't have staff to open another room.*
- *(Nurse) Maybe if we reconsider the lamp-treatment...*
- *(Clinical coordinator) Exactly, and then we hope that the eye baby can go back to his own hospital later in the evening.*

For peer review only

Standards for Reporting Qualitative Research (SRQR)

O'Brien B.C., Harris, I.B., Beckman, T.J., Reed, D.A., & Cook, D.A. (2014). Standards for reporting qualitative research: a synthesis of recommendations. *Academic Medicine, 89(9)*, 1245-1251.

No. Topic	Item
**Title and abstract**	
S1 Title	Concise description of the nature and topic of the study identifying the study as qualitative or indicating the approach (e.g., ethnography, grounded theory) or data collection methods (e.g., interview, focus group) is recommended<br><br>**Qualitative descriptive study is part of the title.**
S2 Abstract	Summary of key elements of the study using the abstract format of the intended publication; typically includes objective, methods, results, and conclusions<br><br>**The 300 words abstract contain objectives, design, participants, results and conclusions**
**Introduction**	
S3 Problem formulation	Description and significance of the problem/phenomenon studied; review of relevant theory and empirical work; problem statement<br><br>**Problem statement on line 84 followed by relevant theory. Study Aim is located at line 100-102.**
S4 Purpose or research question	Purpose of the study and specific objectives or questions<br><br>**The explorative qualitative design is guided by the study aim. A specific area for improvement in indicated on line 97-99**
**Methods**	
S5 Qualitative approach and research paradigm	Qualitative approach (e.g., ethnography, grounded theory, case study, phenomenology, narrative research) and guiding theory if appropriate; identifying the research paradigm (e.g., positivist, constructivist/interpretivist) is also recommended<br><br>**The ethnographic design and participatory observations are described in the methods section, sub headline "Design" line 105-109.**
S6 Researcher characteristics and reflexivity	Researchers' characteristics that may influence the research, including personal attributes, qualifications/experience, relationship with participants, assumptions, or presuppositions; potential or actual interaction between researchers' characteristics and the research questions, approach, methods, results, or transferability

For peer review only

	**The researchers characteristics is mentioned in the methodological bulletpoint on page 3 line 63-64 and further discussed in "Limitations" line 403-405. Trustworthiness is discussed in the last paragraph in "Limitations"**
S7 Context	Setting/site and salient contextual factors; rationale[a]<br><br>**Setting is presented in the *introductions first paragraph* and a subheading under methods "Design and setting" Line 105-109. 110-115.**
S8 Sampling strategy	How and why research participants, documents, or events were selected; criteria for deciding when no further sampling was necessary (e.g., sampling saturation); rationale[a]<br><br>**The sample of data are mentioned in line 131-144. Saturation is not specifically discussed.**
S9 Ethical issues pertaining to human subjects	Documentation of approval by an appropriate ethics review board and participant consent, or explanation for lack thereof; other confidentiality and data security issues.<br><br>**Ethics approval information on line 433.**
S10 Data collection methods	Types of data collected; details of data collection procedures including (as appropriate) start and stop dates of data collection and analysis, iterative process, triangulation of sources/methods, and modification of procedures in response to evolving study findings; rationale[a]<br><br>**Data collection and data sources are presented in the methods section, sub-headline "Data collection" line 131-144.**
S11 Data collection instruments and technologies	Description of instruments (e.g., interview guides, questionnaires) and devices (e.g., audio recorders) used for data collection; if/how the instrument(s) changed over the course of the study<br><br>**Audio recording presented on line 147. Other instruments are not applicable in the ethnographic design.**
S12 Units of study	Number and relevant characteristics of participants, documents, or events included in the study; level of participation (could be reported in results)<br><br>**Not applicable in the ethnographic design, ample data is presented through quotes and appendix 1.**
S13 Data processing	Methods for processing data prior to and during analysis, including transcription, data entry, data management and security, verification of data integrity, data coding, and anonymization/deidentification of excerpts<br><br>**Analysis and collection of data were iterated according to the study design.**
S14 Data analysis	Process by which inferences, themes, etc., were identified and developed, including researchers involved in data analysis; usually references a specific paradigm or approach; rationale[a]<br><br>**Analysis method presented in line 146-161 and "table 1" for further transparency.**
S15 Techniques to enhance trustworthiness	Techniques to enhance trustworthiness and credibility of data analysis (e.g., member checking, audit trail, triangulation); rationale[a]

For peer review only

	**Trustworthiness discussed in line 406-412**
**Results/Findings**	
S16 Synthesis and interpretation	Main findings (e.g., interpretations, inferences, and themes); might include development of a theory or model, or integration with prior research or theory<br><br>**Results is based on an interpretation through a lens of the theoretical concept of resilience. Integration with theory is found in the discussion.**
S17 Links to empirical data	Evidence (e.g., quotes, field notes, text excerpts, photographs) to substantiate analytic findings<br><br>**Extensive quotes and field notes presented in results and appendix 1.**
**Discussion**	
S18 Integration with prior work, implications, transferability, and contribution(s) to the field	Short summary of main findings; explanation of how findings and conclusions connect to, support, elaborate on, or challenge conclusions of earlier scholarship; discussion of scope of application/generalizability; identification of unique contribution(s) to scholarship in a discipline or field<br><br>**Integration with existing management theory for example in line 341-343, 363-367, 369.**
S19 Limitations	Trustworthiness and limitations of findings<br><br>**Trustworthiness and limitations are discussed in lines 392-412**
**Other**	
S20 Conflicts of interest	Potential sources of influence or perceived influence on study conduct and conclusions; how these were managed<br><br>**No competing interests line 442**
S21 Funding	Sources of funding and other support; role of funders in data collection, interpretation, and reporting<br><br>**No specific funding line 444.**

[a]The rationale should briefly discuss the justification for choosing that theory, approach, method, or technique rather than other options available, the assumptions and limitations implicit in those choices, and how those choices influence study conclusions and transferability. As appropriate, the rationale for several items might be discussed together

For peer review only

BMJ Open

A FIRST LINE MANAGEMENT TEAM´S STRATEGIES FOR SUSTAINING RESILIENCE IN A SPECIALISED INTENSIVE CARE UNIT – A QUALITATIVE OBSERVATIONAL STUDY

Journal:	*BMJ Open*
Manuscript ID	bmjopen-2020-040358.R2
Article Type:	Original research
Date Submitted by the Author:	07-Dec-2020
Complete List of Authors:	Hybinette, Karl; Karolinska Institutet Department of Learning Informatics Management and Ethic; Karolinska Hospital, Astrid Lindgrens Childrens Hospital<br>Pukk Härenstam, Karin; Karolinska Institutet Department of Learning Informatics Management and Ethic; Karolinska University Hospital, Paediatric Emergency Department<br>Ekstedt, Mirjam; Karolinska Institutet, LIME; Linneuniversitet,
**Primary Subject Heading**:	Medical management
Secondary Subject Heading:	Emergency medicine, Health policy, Health services research, Health informatics, Intensive care
Keywords:	QUALITATIVE RESEARCH, HEALTH SERVICES ADMINISTRATION & MANAGEMENT, Health policy < HEALTH SERVICES ADMINISTRATION & MANAGEMENT, Organisation of health services < HEALTH SERVICES ADMINISTRATION & MANAGEMENT, Risk management < HEALTH SERVICES ADMINISTRATION & MANAGEMENT

SCHOLARONE™ Manuscripts

I, the Submitting Author has the right to grant and does grant on behalf of all authors of the Work (as defined in the below author licence), an exclusive licence and/or a non-exclusive licence for contributions from authors who are: i) UK Crown employees; ii) where BMJ has agreed a CC-BY licence shall apply, and/or iii) in accordance with the terms applicable for US Federal Government officers or employees acting as part of their official duties; on a worldwide, perpetual, irrevocable, royalty-free basis to BMJ Publishing Group Ltd ("BMJ") its licensees and where the relevant Journal is co-owned by BMJ to the co-owners of the Journal, to publish the Work in this journal and any other BMJ products and to exploit all rights, as set out in our licence.

The Submitting Author accepts and understands that any supply made under these terms is made by BMJ to the Submitting Author unless you are acting as an employee on behalf of your employer or a postgraduate student of an affiliated institution which is paying any applicable article publishing charge ("APC") for Open Access articles. Where the Submitting Author wishes to make the Work available on an Open Access basis (and intends to pay the relevant APC), the terms of reuse of such Open Access shall be governed by a Creative Commons licence – details of these licences and which Creative Commons licence will apply to this Work are set out in our licence referred to above.

Other than as permitted in any relevant BMJ Author's Self Archiving Policies, I confirm this Work has not been accepted for publication elsewhere, is not being considered for publication elsewhere and does not duplicate material already published. I confirm all authors consent to publication of this Work and authorise the granting of this licence.

For peer review only

A FIRST LINE MANAGEMENT TEAM´S STRATEGIES FOR SUSTAINING RESILIENCE IN A SPECIALISED INTENSIVE CARE UNIT – A QUALITATIVE OBSERVATIONAL STUDY

Authors

Karl Hybinette[1,2], Karin Pukk Härenstam[1-3], Mirjam Ekstedt[1, 4]

Affiliation

[1] Department of Learning, Informatics, Management and Ethics, Karolinska Institute, Stockholm, Sweden

[2] Theme Children & Women´s Health, Karolinska University Hospital, Stockholm, Sweden

[3] Paediatric Emergency Department, Karolinska University Hospital, Stockholm, Sweden

[4] Department of Health and Caring Sciences, Linnaeus University, Kalmar, Sweden

Corresponding author

Karl Hybinette

Karolinska Institute

Department of Learning, Informatics, Management and Ethics,

Tomtebodavägen 18 A

SE 171 77 Stockholm

E-mail: karl.hybinette@ki.se

Phone: 0046704297173

For peer review only - http://bmjopen.bmj.com/site/about/guidelines.xhtml

ABSTRACT

Objectives

Acute care units manage high risk patients at the edge of scientifically established treatments and organisational constraints while aiming to balance reliability to standards with the needs of situational adaptation (resilience). First line managers are central in coordinating clinical care. Any systemic brittleness will be evident only in retrospect through for example care quality measures and accident statistics. This challenges us to understand what successful managerial strategies for adaptation are and how they could be improved. The managerial work of balancing reliability and adaptation is only partially understood. This study aims to explore and describe how system resilience is enhanced by naturally occurring coordination performed in situ by a management team under variable circumstances.

Design

An explorative observational study of a tertiary Neonatal Intensive Care Unit (NICU) in Sweden. One year of broad preparatory work followed by focused shadowing observations of coordination analysed through inductive-deductive content analysis from a perspective of resilience engineering.

Participants

A team of managers (i.e. clinical coordinators, head nurses, senior medical doctors).

Results

The results describe a functional relationship between the NICU´s level of manoeuvrability and a progression of adjustments in the actual situation, expressed through recurring patterns of adaptation. Managers focused on maintaining coherence in escalating problematic situations by facilitating teamwork through goalsetting, problem-solving and circumventing the technical systems’ limitations.

Conclusions

For peer review only - http://bmjopen.bmj.com/site/about/guidelines.xhtml

Coordination supports a coherent goal setting by increased team collaboration and is supported by team members´ abilities to predict the behavior of each other. Our findings suggest that in design of future research or training for coordination, the focus of assessment and reflection on adaptive managerial responses may lie on situations where the system was "stretched" or "needed reorganization" and that learning should be about whether the actions were able to achieve the short-term goals while preserving the long-term goals.

Keywords

Qualitative research, Health services administration & management, Health policy, Organisation of health services, Risk management

Wordcount

4877

Strengths and limitations of this study

- The explorative ethnographic design allowed for a deeper understanding of the underspecified (hidden) work of first line managers.
- Several iterations of data collection and analysis allowed for an initially wide and later more focused data collection that opened opportunities for the researchers to follow up on specific findings.
- The inductive-deductive analysis allowed the researchers to follow and describe patterns of recurring codes within the whole dataset.
- The first author (KH) who conducted the fieldwork is an experienced neonatal intensive care nurse which may have affected the interpretations drawn in this study.
- The explorative study design was suitable for describing a complex process without measurable clinical outcomes.

For peer review only - http://bmjopen.bmj.com/site/about/guidelines.xhtml

BACKGROUND

The neonatal intensive care unit (NICU) is a specialized intensive care unit that serves as its own emergency department, intensive care unit and a pre- and postoperative ward, requiring a wide array of interrelated multi-professional teams to operate in a coordinated fashion. The many roles of the unit are coupled with the pressures of maintaining high-quality and individualized care, family support and safe care practices. Some situations are manageable by reorganizing teams internally while others require cooperation with external units.

The NICU is distributed over three hospitals in an integrated network of independent units organized under one head of department. Daily operations are overseen by the strategic operations manager to ensure collaboration for overall performance across the three units. The overarching goals as understood by the authors are individualized care, family support, high quality care and safe care practices (Figure 1).

[Insert figure 1 here]

Maintaining quality and safety is a persistent problem considering acute admissions, staff shortage and rapidly deteriorating patients with life-threatening conditions (1,2). Safety is often defined as one of many variations of ‘the absence of preventable harm to a patient during the process of health care’ (3). During the last decades of patient safety research a movement towards an inclusive view of supporting the healthcare systems´ ability to sustain required (normal) operations has emerged, the resilience engineering (RE) perspective (4). An important point made within the RE perspective is that safety can be enhanced by a combination of structure and control on one hand and adaptations on the other (5).

For peer review only - http://bmjopen.bmj.com/site/about/guidelines.xhtml

Healthcare is to be understood as a complex sociotechnical system (6). It is not only defined by the boundaries of physical locations (7). People with their skills and relationships, rules, regulations and even work place culture may impact system performance (6). Research on resilience in healthcare suggest that successful as well as unsuccessful outcomes emerge from the fluid arrangement of system components (coordination) (8). Coordination is defined as "*…the deliberate and orderly alignment or adjustment of partners' actions to achieve jointly determined goals"* (9). 'Partners' in the healthcare setting are those who share a common goal of delivering care to patients. People who perform coordinative work are a part of a "*process by which team resources, activities, and responses are organized to ensure that tasks are integrated, synchronized, and completed within established temporal constraints"* (10).

Although coordination is an integral part of everyday healthcare management we know very little about how *managers* learn the strategies they use and which of the work practices they develop that are successful (11,12).

This study aims to explore how system resilience is enhanced by naturally occurring coordination performed in situ by a management team (i.e. clinical coordinators, head nurses, senior medical doctors) under variable circumstances.

METHODS

Design and setting

This study uses an explorative ethnographic design using participatory observations and an abductive approach to capture and analyse naturally occurring coordination in situ (13). The reason for focusing on action-interaction was to capture a deep understanding of the varying conditions under which

For peer review only - http://bmjopen.bmj.com/site/about/guidelines.xhtml

decisions and coordination took place (14). The Standards for Reporting Qualitative Research (SRQR) checklist was used to improve the transparency of research (15).

The study took place in a tertiary level NICU with an approximate capacity of 70 cots divided over three wards located in separate hospitals. The patient mix of the three wards is dependent on local factors such as the size and risk profile of adjacent delivery wards and the availability of paediatric surgical capacity. Staffing for the high acuity patients is normally one nursing team per two patients (one nurse and one assistant nurse). Paediatricians and neonatologists are allocated over the three wards depending on availability and competence.

Each of the three wards is managed by a clinical coordinator, a head nurse and an operations manager during daytime (Figure 1). The clinical coordinator performs tasks such as rostering, planning for patient flows (admissions, discharges and transports) and does clinical work when needed. The head nurse is formally responsible for the work environment and quality of care. The operations manager is a senior neonatologist with an overall responsibility for the medical quality and patient flow. A strategic operations manager has mandate to move patients between hospitals within the own organisation or to hospitals outside the region. All managers are clinical specialists (nurses and neonatologists). Hereafter we will refer to this team as the management team.

Data collection

Data was collected between January and February 2017. The head of the department gave permission to conduct participant observations at the unit. All staff was informed about the procedures in a staff meeting and they were informed that they could decline from being observed at any time according to the ethics approval. The clinical coordinators (four individuals) gave written consent to being shadowed

For peer review only - http://bmjopen.bmj.com/site/about/guidelines.xhtml

after receiving written and oral information. The coordinators were all women, experienced nurses with more than 10 years of NICU experience.

Data collection was structured in iterative cycles of collection and analysis, starting with descriptive observations to get familiar with the work environment and relevant aspects of the managers' work (16). The descriptive observations focused primarily on ´places´ were the coordination of work was apparent, for example at the head nurses and secretaries open office area and at the management teams´ office at the center of the ward. The intermittent recording and analysis of field notes during initial observations yielded research questions that were in focus during the following observations.

The focused observations targeted selected situations such as rostering for the next shift, start-up meetings and handovers between shifts in addition to shadowing of coordinators. Data was collected through ad-hoc interviews with staff and managers during or after the shadowing (16). Shadowing meaning "following people, wherever they are, whatever they are doing" (17). Artefacts, including coordinators' notes on patients' medical status, occupancy- and rostering charts were copied and collected. All relevant aspects of the environment were captured in field notes during or after the observations, along with researcher's memo-writing over personal reflections and thoughts about what was happening.

Analysis

All meetings and interviews were audio recorded and transcribed verbatim. Transcriptions from tape-recorded dialogues were placed in a correct temporal order along with the field notes, so that the mix of field notes and transcription chronologically represented the full workday. Transcripts were read through several times, followed by discussions in the research group about the level of detail in the data and reflections KH had regarding the observed work shift (18). The initial inductive analysis went

For peer review only - http://bmjopen.bmj.com/site/about/guidelines.xhtml

151 through a two-step process (columns two and three in Table 1). First, a conversation or a situation of relevance for the study's aim (a meaning unit) was selected and the question "What is happening here?" was directed to the data. Next, the selected conversation or situation was analysed in the context of the entire scene where it took place, with the question "Why or how is it happening here?". The interpretation was condensed and labelled with one or several codes (Table 1). The codes and their relations were frequently discussed in the research team and sorted into tentative sub- and main categories. Field notes were included in the analysis as means to reflect on the researchers' pre-understanding of the context. Moving back and forth between induction and deduction was a way to discover meaningful underlying patterns that made it possible to integrate concrete behaviour and deep contextual structures. Lastly, a deductive comparison of interdependencies between the main categories and sub-categories in relation to the theoretical concept of resilience was performed (14).

review only

For peer review only - http://bmjopen.bmj.com/site/about/guidelines.xhtml

Table 1 Example of the analysis in two-steps: going from *raw data* to *interpretation of incident* and to *analytical interpretation in context*, *code* and *memo*.

Raw data	Interpretation of incident	Analytical interpretation in context	Code	Memo
The observation begins in the flow room. The flow room is a small room with two workstations where the coordinator has her seat. There are several information sources hanging on the walls and post-it notes on the computers. The coordinator meets the observer in the flow room after having walked around the ward to check all the patient rooms.	In the physical environment, several different communication tools are gathered within a small area, i.e., tools used to summarize, remember, and disseminate important information. Information exchange occurs on paper notes stuck to computers, through software, and when the coordinator herself walks around the ward.	Information exchange is one part of coordination and can be performed through predefined channels and tools, but also more intuitively through physical meetings within the ward while the manager compares notes to what is experienced. The environment has been adapted for having several different information channels intersecting in one place.	Adapting environment – cluster tools for facilitating information exchange	The coordinator has gathered information about the current situation in the patient rooms by walking around and taking a look. She then looks at the system level, occupancy lists, and information from the other coordinators. If coordination means exercising control, real life information gathering is probably an important step.
The coordinator sits down at a computer and begins counting patients on her paper copy of the occupancy list and in TakeCare [electronic health record]. She reports the occupancy in Belport [national occupancy chart] and talks a bit about this.	Counting patients and manually entering the number into the national occupancy report is one of the first things the coordinator does in the morning.	The coordinator gets an idea of the status and distributes it to the rest of the country as information. This is proactive management, as future coordination may become easier if reference can be made to Belport, or if you know that the other units in the country your information.	Information handling	Reporting your status is a way of exercising control by impacting others perception regarding the degrees of freedom at your end.

PATIENT AND PUBLIC INVOLVMENT

Public and patient involvement was not applicable in this research.

For peer review only - http://bmjopen.bmj.com/site/about/guidelines.xhtml

RESULTS

The analysis resulted in six subcategories and two unifying main categories (table 2).

The ´how´ and ´when´ of coordination is presented in Table 2 that illustrate the functional relationship between the two main categories *adjustments to meet the actual situation* and *maneuverability* with their respective sub-categories. The sub-categories *Supporting system cohesion* and *Extending system boundaries* describe the joint work of managers and clinical staff. *Adapting the structure and roles of the coordinating management team* and *Shifting between information sources for better sensemaking* describe the management team's internal work.

Extracts from the field notes and conversations are presented to clarify the findings. More comprehensive material is provided in Appendix 1.

For peer review only

Table 2 The main category on the y-axis are *adjustments to meet the actual situation* and on the x-axis; *maneuverability.* Four sub-categories on the y-axis describe strategies (i.e. the work that managers do); and the three sub-categories on the x-axis describe a progression of a perceived level of manoeuvrability based on the expressed availability of *degrees of freedom* with illustrative quotes.

		**Manoeuvrability**		
		**Losing control – reorganisation**	**Stretching the system to work outside ordinary conditions**	**Everyday work under ordinary conditions**
**Adjustments to meet the actual situation**		”So [the other hospital] have 20 babies now, seven acute. They have opened a temporary room but have no staff for it. We have no transport team [to use as staff] because they are on their way to [another city] […] I still won't send babies to other counties, which we can't because we have no transport team available […] either we find some staff here that can go there or we have to order two on overtime. How do we usually do?” (Operations manager).	“We have put those two [patients] together in 9:1 to get an emergency cot in 9:2 for the twins [9 refers to a room divided in two sections equipped for one patient per section 9:1 and 9:2], I told the father that they may have to move out. But then we will be in the situation where they maybe… They may need… That father is very new. So, I think that in that case they will have to be two [staff] in there too.” (Clinical coordinator). [Q1 – appendix 1]	”Oh, so we are only at eight intensive care babies and three in family rooms. So, it's a pretty good situation. And we have nothing acute in the delivery- or antenatal wards. I just checked.” (Operations manager).
	**Supporting system cohesion**	• Delay work and evaluate the situation<br>• Isolate problems and focus on re-creating manoeuvrability.<br>• Exploit possibilities of extraordinary individual achievements (trade-off individual resilience for system control).	• Goalsetting towards protecting manoeuvrability.<br>• Goalsetting for promoting basic safe care practices at the clinical level through minimal staff allocation and skill mix.<br>• Sacrificing continuity in patient assignments for saving lives.	• Goalsetting towards family-centred care<br>• Goalsetting of individualised care.<br>• Managing optimal staff allocation for maintaining professional development and education.<br>• Controlling occupancy and redundant capacity through predefined strategies.<br>• Monitoring state of the ward at the clinical level by regular walkarounds in the clinical work environment.
	**Extending system boundaries**	• Identifying novel use of any existing external resources (i.e. use of paediatric emergency transport team and other wards).<br>• Shedding managerial tasks for participating in clinical emergency work (trade-off management for clinical work).	• Managing occupancy trade-offs between facilities and staffing (higher occupancy in fewer rooms lowers staff requirements).<br>• Utilising individual managers social networks within predefined limits for proactive problem-solving.	• System working within normal boundaries.
	**Adapting the structure and roles of the coordinating management team**	• Make *loss of control* explicit in the management team.<br>• Moving from understanding the situation to making rapid decisions close to the clinical level.	• Relaying information on the patients' clinical situation to mid- and upper-level managers (information priority bottom-up).	• Relaying high level plans to clinical level workers through regular briefings (information priority top-down).<br>• Participate in regular medical discussions on patients' status´.
	**Shifting between information sources for better sensemaking**	• Dropping computerised aides, rostering systems and staffing charts for handwritten notes and memos.<br>• Using face-to-face communication with people in close vicinity (shedding electronic communication).	• Seeking ad-hoc meetings within the management team for calibrating information of the situation and possible workarounds.<br>• Verbally explaining situations to other managers.	• Regular use of computerised systems and handwritten notes.

Manoeuvrability

For peer review only - http://bmjopen.bmj.com/site/about/guidelines.xhtml

Managers face situations where they must balance a limited number of staff and demands for high occupancy with the task of supporting clinical teams in their care of patients. Unpredictable factors such as acute admissions, staff availability and the medical progression of patients put managers in situations where they might quickly have to re-adjust and re-plan, often ad-hoc with scarce information of the overall situation. Part of the observed dialogue relate to the management team's efforts to identify alternative ways forward. See quotes at the head of Table 2. Further examples of the choices made under each sub-category of manoeuvrability is presented in table 3.

In the quote below, the head nurse acknowledges that the inflow of emergency patients is sometimes impossible to avoid.

"The patient flow, do you participate in that?" (Observer). "No, no more than that I can say ´stop´, because I don't have the staffing." (Head nurse). "Ok, so you can say that too?" (Observer). "Mm, I can say that I have five or six teams. But…" (Head nurse). "But you cannot say stop today." (Coordinator). "No, but it is like that. Even if we say stop the babies are being born. And we have to take care of them." (Head nurse).

Table 3 Examples of ´situations´ and ´choices made´ sorted under each sub-category of manoeuvrability.

Losing control – reorganisation	Stretching the system to work outside ordinary conditions	Everyday work under ordinary conditions
**Situation with several problematic trade-offs.**<br>The coordinator approaches the head nurse when she cannot find a suitable cot for a new patient (Field-notes).<br>"We have another problem." (Coordinator). "Right, what?" (Head nurse). "This new week 28 [patient], I cannot put in room 10 [a room with four cots, one is available], the other baby in there is terminal and about to die." (Coordinator). "But if we place him in room 16 or 15?" (Head nurse). "No, I can't put it in 15 because I have two cots in there for the twins and they must be together [also incoming patients]." (Coordinator). […] "We cannot have the twins remaining at the stabilisation unit at delivery, there might be another patient coming in." (Coordinator). "Okay, because I thought it	**Exploring options to avoid patient transfers because of staff shortage.**<br>"NN [strategic operations manager] think that we can conserve personnel by moving ´K´ [a patient] to ´Ts´ room [another patients]." (Head nurse). "Okay." (Observer). "But I am not so sure we conserve personnel by that; and the family have already moved several times, you know that they are the ones that was supposed to be moved to [another hospital]." (Coordinator). "Okay" (Observer). "It [the patient] started to have breathing problems and came back. And now after moving back here they will have to move again ´sigh´." (Coordinator).	**The deliberate adaptation of an administrative routine.**<br>"I have talked to NN [a nurse currently placed at the emergency stabilisation unit in the delivery ward] because there is a week 35 baby there that the doctor did not want to take it up to the ward during the nightshift." (Coordinator). "No?" (Observer). "Yes, then he has of course been there a little longer that his allowed six hours." (Coordinator).

For peer review only

For peer review only - http://bmjopen.bmj.com/site/about/guidelines.xhtml

would be a better idea to move the twins directly from delivery [to another hospital] if they need to move anyway at some point." (Head nurse). "Mm, but where should they move. All hospitals are full [in the county]. We would have to move them out of county." (Coordinator). "Yes, we will never be allowed to do that." (Head nurse).		

Everyday work under ordinary conditions

The management team have planned meetings; they sweep the ward to collect information on the state of things and relay high level plans to workers.

The coordinator begins her shift by conducting a walkaround of the ward, saying hello to the nurses and doctors as she is asking if everything is all right. After the walkaround she sits down to begin exploring her staff roster and patient occupancy charts (Field-notes).

Under ordinary conditions there is a minimal observable need for managers to manually adapt information they extract from technical systems, regarding for example occupancy, patient acuity levels and staffing. Managers describe how their experience of the technical systems' limitations are learned on the job and how workarounds are taught between individuals.

The coordinator makes a note of something on her occupancy chart (Field-notes). "What did you write on that chart?" (Observer). "CT 11:15 [Computer Tomography at 11:15]." (Coordinator). "And it is used for that kind of information?" (Observer). "I print one of these charts because there is more space to write on. I think… I think many… I think NN and NN [two other coordinators] use this too. And NN [another coordinator] use it because I taught her. And I use it because NN [a coordinator who quit earlier] taught me." (Coordinator). "Okay, that is nice. But is it the same information you all use it for?" (Observer). "Well, I don't know?" (Coordinator). "Not important?" (Observer). "Well, I don't think so, I use it for… those that are planned for other hospitals, delivery ward, antenatal… Oh! I forgot to write that today [makes another note on the chart] and stuff that I need to do." (Coordinator).

For peer review only - http://bmjopen.bmj.com/site/about/guidelines.xhtml

Stretching the system to work outside ordinary conditions

The following situation is a typical example of how the management team must re-organize and adapt to stretched conditions. The ad-hoc meeting in the example illustrates that strategic goals of family centred care and optimal staff allocation are traded for immediate medical priorities, basic safe care practises and protection of the wider system. Other patients on the ward are cared for by their respective teams, avoiding exposure of this emerging crisis because it is handled by the paediatric emergency transport team.

The plan for this day is to admit one intermediary level patient that was born during the night and is waiting for a cot at the NICU. One patient has been diagnosed with a multi-resistant bacterium and will need increased hygiene standards (Field- notes) […] "We are plenty of people today, which is nice. But when NN [strategic operations manager] asked if we had a lot of capacity, I had to say no." (Clinical coordinator). […] "...one important thing. This baby that needed eye surgery is now acute and will arrive soon. They will land directly in the operating room and the transport team will take care of the baby until it can go back home to its own hospital. (Operations manager). [Q2 – Appendix 1]

The plan for postoperative care after acute eye-surgery was for the patient to be assigned a cot and a nursing team on the ward. The situation was managed by using the transport team to temporarily care for the baby inside the operating room until it was stable enough for transport to another hospital. This decision had possible implications for the whole management team. The clinical coordinator wanted to know about the utilisation of staff and facilities. The head nurse about workplace safety, quality of care and economy. The operations manager had responsibility for the medical quality and the strategic operations manager for the possibility of helping one of the other wards with staffing.

Losing control – reorganisation

For peer review only

The category of ´losing control´ was characterised by managers shedding managerial tasks for participating in clinical emergency work, isolating problems and focusing on re-creating manoeuvrability. The focus was to protect clinical teams from being exposed to the rapidly shifting plans and priorities at the managerial level. Strategies for regaining control can be to sacrifice the goal of continuity by transferring at-risk mothers to other hospitals (deferral), or to temporarily transfer additional weight of medical care to neighbouring sub-systems such as the paediatric emergency transport team or the managers themselves.

The following situation illustrates a reorganisation at the verge of losing control. The coordinator tried to reserve an empty cot for possible additional acute emergency admissions.

“This is not good, its full [the ward]. We have no space when this eye baby arrives.” (Clinical coordinator). The clinical coordinator walks to the room where a nurse oversees the twins that were supposed to be transported out the next day but are now showing symptoms of infection. (Field- notes). “Do you think these twins could be together in a twin cot?” (Clinical coordinator) […] “Well, I don´t know. This one is just on the margin of managing without incubator.” (Nurse) […] “The problem is that I don’t have staff to open another room.” (Clinical coordinator). [Q3 – Appendix 1]

The manager handled the situation by putting twins together in one cot, thereby utilising one nurse to care for three babies which is more than the goal of two babies per nurse. This manoeuvre created an opportunity to temporarily handle five patients (with one empty emergency cot) in a room with staffing for four. The nurse expressed concern for her patient but remained focused on finding a solution.

When the managers started shedding managerial tasks for bedside operative work, they risked losing the ability to meet other managers and keep up to date with the ward. Management decisions in these situations were then based on a narrower understanding of the bigger picture. Computer aides were less used (or not at all) because of their inability to present rapidly changing borderline conditions.

For peer review only - http://bmjopen.bmj.com/site/about/guidelines.xhtml

Adjustments to meet the actual situation

It was observed how the mandate for making decisions was distributed across the management team and clinical nursing teams (speech bubbles in figure 2). The clinical coordinator moved freely across the unit and made independent decisions regarding resource allocation, rostering and whether to shed her own managerial work to help the clinical teams. The clinical coordinator also provided the other managers with information when they needed to adjust their plans.

[Insert figure 2 here]

Supporting system cohesion

Members of the management team were observed to often communicate through ad-hoc meetings where verbal information was compared to patient rosters, patient conditions, staffing and workload indicators. They worked close to the clinical context and discussed the current goals' attainment for individual clinical teams and what the current trade-offs were. The managers compared computer- and paper-based notes, as well as information provided at start-up meetings to update themselves of the status of the ward and where the situation was heading (umbrella perspective in Figure 2).

The coordinator is back in room nine to alleviate the staff for lunch. She checks in on the baby that has been in acute surgery for intestinal obstruction. There are lots of beeping sounds, but no one seems alarmed. The head nurse enters the room and seeks the coordinators attention, but initially fails as the coordinator is tending to a patient. Eventually the coordinator looks up (Field-notes). "Didn't they [the surgeons] say already this morning that this patient was up for re-operation?" (Coordinator). "After lunch apparently. Then what do we do with NN [another patient]?" (Head nurse).

In the example above the managers discussed how two patients' trajectories were affected by the surgeons rescheduling. The management team negotiated situations that needed simultaneous

For peer review only

For peer review only - http://bmjopen.bmj.com/site/about/guidelines.xhtml

attention, like prioritizing readiness and clinical capacity in some parts of the ward while maintaining family centered care and staff education in others (Illustrated as the three clinical teams in Figure 2).

Extending system boundaries

The need for extending the system's boundaries emerged as the pressure of prioritizing decisions increased. The management team made use of other units' facilities or staff, like delaying patients in the operating theatre or letting a transport team care for the patient for some time before handing it over to the regular staff. The management team utilised auxiliary staff and overlapping competencies of different professional groups. Sometimes the managers themselves doubled as clinical staff within their vocation.

"It is already a little tight if it arrives [the surgery baby], right now we have low workload in room nine and ten with four patients and the other goes back to [another hospital out in another region] at five [in the afternoon]. We hope. But it is also, I mean, if there is an acute admission and he... I do not think NN [a nurse] can have four patients by himself out there. Since the father needs quite a lot of help." (Coordinator). "They are really good patients those babies" (Operations manager). "What about the midwives then? [that belongs to the adjacent delivery ward]" (Coordinator). "There are four of them [turns to face the operations manager]?" (Head nurse). "Yesterday someone said that you can have four patients by yourself." (Operations manager). "Not by yourself" (Coordinator). "With an assistant nurse." (Head nurse). "Yes with an assistant yes, that is okay but." (Coordinator). "So there are three patients left?" (Head nurse). "And then we have put two in the same cot at 9:1 [bed one in room nine]." (Head nurse 2). "Okay." (Head nurse).

Adapting the structure and roles of the coordinating management team

For peer review only - http://bmjopen.bmj.com/site/about/guidelines.xhtml

The management team fluently adapted its own structure in situations where there were not enough resources to manage within everyday routines, when there was urgency or when some of the management team members were not available with their specific expertise and mandate. This structural adaptation was observed when individuals in the management team shifted from relaying plans from top-down to working with patients and gathering information from bottom-up.

"In room nine is that week 22 baby that came in yesterday. They are intubating now so they use a lot of people. There were no head nurses here at seven, so I decided myself that NN [nurse] got to be alone at the stabilisation room [at the delivery ward]. The doctors there have to work a little harder now." (Clinical coordinator).

Shifting between information sources for better sensemaking

As the situation on the ward became more complex the management team increased their reliance-on handwritten notes rather than the standard computer-generated lists for staffing and patient acuity information.

The coordinator used handwritten notes as memory aides in team discussions. The notes were mainly short markings, phrases, or single words in the margins like "discharge planned" or "need antibiotics". The limitations of computer-generated patient rosters to convey this type of information on real world complexities were expressed by members of the management team.

The national occupancy chart for example was only able to classify patients as high or low acuity without regards of other factors. When there was a need to work outside the binary world of two patient groups, the team stopped using this computer-generated aide and instead relied on their own domain knowledge, personal network and the stability of the management teams' understanding of the bigger picture.

For peer review only - http://bmjopen.bmj.com/site/about/guidelines.xhtml

"Well and this is actually look correct [number of beds in the national occupancy chart] 14 in total. But with the stabilisation beds. That makes it 12 here plus two there… But it is also a little [inaccurate]. Because then you calculate [all of them as intensive care beds]. The stabilisation beds are supposed to be low acuity". (Clinical coordinator)

The following quote illustrates what happens when the computerised information indicated normal occupancy when the off-going night nurse reported understaffing for the same shift.

"How did this happen?" (Clinical coordinator).

"Well, because NN [one of the nurses] who is work-training after sick leave was included in the staffing. The parenthesis was probably put there later [points at the handwritten parenthesis in the rostering folder, indicating that NN should not be included in the staffing]". (Night nurse)

DISCUSSION

The management team in this study exhibited a range of mindful adaptations for sustaining the units´ capacity for expressing resilience. Such as sacrificing low level goals based on up-to date information and continuous assessments of what would be minimally intrusive for the overall performance of the system (figure 2). Our findings elucidate a link between adjustments to meet the actual situation and the available manoeuvrability of the system.

Supporting coherence

The management team aimed to balance the demands and capacity of multiple teams that operate in separate rooms while tending to patients with a wide variety of problems and acuity. For practical reasons the teams could not always meet to communicate with each other. A defining characteristic of a

For peer review only - http://bmjopen.bmj.com/site/about/guidelines.xhtml

multiteam system is the ability of component teams to modify individual goal hierarchies while sharing a common distal goal or set of goals (12). During the observations the management team and the clinical staff consequently agreed on making provision of acute care to rapidly deteriorating patients a top priority, allowing us to identify it as a core mission (i.e. purpose of the system) (19). Other priorities were more likely to be put on hold and resumed later or to be permanently dropped.

When the care teams were unable to communicate, maintaining coherence was an important factor for the managers´ maintenance of an umbrella perspective i.e. what they needed in order to understand how the bigger picture of their interventions fit together (20). At this point, we can say two things about coherence, with implications for the training of managers as adaptive teams (21). First, that coordination supported a coherent goal setting with increased team collaboration and second, that it was enhanced by team members' ability to predict the most likely priorities of each other.

Reorganising to support manoeuvrability

Everyday work of the management team was characterised by seamlessly and actively organising and reorganising. Our observations illustrate how the management team made use of early investments in for example staff´s expertise, deep domain knowledge and the workplace culture to maintain a unit wide focus on the core mission (22). Allowing the care teams to adapt their goals individually exemplifies that being resilient is to be part of a process of identifying conflicting goals in a complex, intractable environment using *"numerous indicators in a proactive fashion to probe a system's adaptive capacity before system-wide collapse results in disaster"* (21). A realisation from studying the management team was the shapelessness of the organisation. We could not observe a formal agenda for how and why the management team was supposed to prioritize in terms of goal achievement below the core mission. Our study suggests that it is up to the management team to support the system by using experience, professional ethos and domain knowledge to negotiate the way forward in a manner

For peer review only - http://bmjopen.bmj.com/site/about/guidelines.xhtml

that resembles the fifth principle of managing the unexpected, as described by Weick and Sutcliffe (23). Specifically, because the flexible decision structures enabled resilient performance when expertise and experience outranked formal hierarchical positions.

Important for the application of resilience in everyday clinical work was the link between adaptation and outcome (successful or unsuccessful) as described in for example the CARE model for researching resilience in healthcare (24). An adaptation is a deviation from work as planned, and it is not always clear beforehand whether the outcome of an adaptation constitutes success or failure in terms of quality and safety. Our study describes managers' adaptive responses to the conflicting demands of acute patient care on one hand and long-term strategic demands on the other (measured as for example respirator days, patient throughput and hospital acquired bloodstream infections).

Balancing between long- and short-term goals

Resilience depended on the use of earlier investments in 'potential opportunities for action' previously described as degrees of freedom. However, low-level goal-sacrifices do represent a loss of potential future degrees of freedom if it is overexploited. In the context of the NICU, families are less prepared for discharge if they are not trained, staff might receive less time learning from experienced colleagues if they do not work together and formal routines might erode if they are not employed.

The balancing act between seemingly irreconcilable goals makes it impossible to decide in retrospect whether coordination was good or bad for the total outcome of the system. Each decision to suspend or sacrifice a low-level goal has implications for the organisation's future capacity for expressing resilience. All teams worked towards the core mission of providing acute care. However, the maintenance of long-term investments was achieved by managing a diversity of low-level goal-sacrifices between many clinical teams (i.e. sacrificing patient education in one and staff education in another). Our findings

For peer review only - http://bmjopen.bmj.com/site/about/guidelines.xhtml

suggest that in design of future research or training for coordination, the focus of assessment and reflection should be on adaptive managerial responses in situations where the system is ‘stretched’ or ‘in need of reorganization’ (table 2). Furthermore, learning should be about whether the actions lead to achievement of short-term goals while preserving the long-term goals.

Limitations

This was a single centre study of a specialised unit with a specific patient clientele that cannot be cared for by any other type of unit available. It was expected that the unit´s high tempo and specialisation would promote a particularly observable coordinative work with the risk that it might introduce the argument of limited transferability to other areas of healthcare. We believe however that the networked structure of three wards is not unique. The specific unit for study enabled us to capture and understand the subtleties of everyday work of first line managers. Further studies are needed to investigate how much of this work may be specific to organizations of both similar and contrasting types.

Using a qualitative cross-sectional design, this study does not allow us to define successful or unsuccessful outcomes. Resilience is described based on the actions taken and further studies are required to operationalise and test our results.

The first author (KH) who conducted the fieldwork is an experienced neonatal intensive care nurse with experience from the studied NICU. The familiarity with the specific type of work may have affected the interpretations drawn in this study.

Trustworthiness during data analysis was addressed by regular peer-check and in seminars with the wider research group and member check. The final analysis was individually validated with the coordinators (25). The iterative process of data collection and analysis was intended to ensure that the

For peer review only - http://bmjopen.bmj.com/site/about/guidelines.xhtml

409 analysis included more than one researcher's interpretation. Transferability was addressed by leaving an audit trail of extracts from the data in the report so that readers from other fields can evaluate if the results are transferable to their respective contexts (26). Table 1 of the methodological section provides a trail of how interpretations of data were made.

413 The use of voice recordings of meetings and conversations was limited to situations where verbal consent could be obtained from all participants, unless explicitly asked not to by any of the participants (27). In larger groups, where participants attended only partially this was not a feasible option and handwritten notes were taken, video recordings were not possible because of difficulties with assuring patient anonymity in the clinical context.

418 CONCLUSIONS

419 We are only beginning to understand managers' strategies for prioritising and acting on the variability of degrees of freedom. Our findings suggest that managers at the clinical level, while being central to the system's capacity for expressing resilience, do not have an explicit model or training for how they approach their work. Furthermore, managers lack the aid of tailored decision support systems. This could depend on well described challenges for design of such interventions (28). It is important that healthcare policy and organisational redesign initiated at higher levels are well calibrated with the nature of managerial work on the clinical level before interventions can be developed. Our recommendations for future research and policy making is to prioritise definition and operationalisation of successful coordination in a wide variety of contexts.

For peer review only - http://bmjopen.bmj.com/site/about/guidelines.xhtml

LIST OF ABBREVIATIONS

RE: Resilience Engineering;

NICU: Neonatal Intensive Care Unit

DECLARATIONS

Ethics approval

This study was approved by the regional ethical review board of Stockholm (2016/1832-32).

Consent for publication

Not applicable.

Availability of data and material

Given that the data is in Swedish and the phrasing of the consent obtained from participants, complete raw data are not available for public sharing. Partial datasets used and/or analysed during the current study are available from the corresponding author on reasonable request. A selection of translated quotes is supplied in the results and methods sections and in appendix 1.

Competing interests

The authors declare that they have no competing interests.

Funding

The authors received no specific funding for this work.

Author Contributions

For peer review only

The study was initiated by KH. The study design was developed in collaboration within the research team. KH performed the data collection. The analysis and interpretation of data was conducted in close collaboration between KH, ME and KPH. KH wrote the first draft of the paper. All authors contributed with writing, critical revisions and approval of the final version.

Acknowledgement

Thank to Professor Eric Hollnagel that contributed with theoretical expertise on the interpretation of data; Professor Richard Cook for valuable discussions about system safety and methodology in the early phase of the study. Thanks also to the participating Neonatal unit.

References

1. Samra HA, McGrath JM, Rollins W. Patient safety in the NICU: A comprehensive review. J Perinat Neonatal Nurs. 2011;25(2):123–32.

2. Bondurant PG, Nielsen-farrell J, Armstrong L. The Journey to High Reliability in the NICU. J perinaltal Nurs. 2015;29(2):170–8.

3. Kim L, Lyder CH, Mcneese-Smith D, Leach LS, Needleman J. Defining attributes of patient safety through a concept analysis. J Adv Nurs. 2015;71(11):2490–503.

4. Wears RL, Hollnagel E, Braithwaite J. Resilient Health Care, Volume 2 : The resilience of everyday clinical work. Farnham: Ashgate Publishing Limited; 2015. 328 p.

5. Eurocontrol. From Safety-I to Safety-II: A White Paper. Netw Manag. 2013;1–32.

6. Braithwaite J, Churruca K, Ellis LA, Long J, Clay-williams R, Damen N, et al. Complexity Science in Healthcare – Aspirations, Approaches, Applications and Accomplishments: A White Paper. Australian Institute of Health Innovation, Macquarie University. Sydney; 2017.

For peer review only - http://bmjopen.bmj.com/site/about/guidelines.xhtml

7. Cilliers P. Boundaries, Hierarchies and Networks in Complex Systems. Int J Innov Manag [Internet]. 2001;05(02):135–47. Available from: http://www.worldscientific.com/doi/abs/10.1142/S1363919601000312

8. Cook RI. Taking Things in One's Stride: Cognitive Features of Two Resilient Performances. In: Hollnagel E, Woods DD, Leveson N, editors. Resilience Engineering: Concepts and Precepts [Internet]. Burlington: Ashgate; 2006. p. 205–21. Available from: http://qualitysafety.bmj.com/lookup/doi/10.1136/qshc.2006.018390

9. Gulati R, Wohlgezogen F, Zhelyazkov P. The Two Facets of Collaboration: Cooperation and Coordination in Strategic Alliances. Acad Manag Ann [Internet]. 2012;6(1):531–83. Available from: http://search.ebscohost.com/login.aspx?direct=true&db=bth&AN=77495192&site=ehost-live&scope=site

10. Cannon-Bowers J, Tannenbaum S, Salas E, Volpe C. Defining team competencies: Implications for training requirements and strategies. In: Guzzo R, Salas E, Associates, editors. Team effectiveness and decision making in organizations. San Francisco, CA: Jossey-Bass; 1995. p. 333–80.

11. Cook R, Woods D. The messy details: Insights from technical work studies in healthcare. In: Proceedings of the human factors and ergonomics society 47th annual meeting. 2003. p. 2002–3.

12. Mathieu J, Marks MA. Multiteam Systems. In: Anderson N, Ones D, Sinangil HK VC, editor. Handbook of Industrial, Work & Organizational Psychology. London, England: SAGE Publications; 2001. p. 289–313.

13. Silverman D. Interpreting Qualitative Data: Methods for Analysing Talk, Text and Interaction. 2 nd. London, England: SAGE Publications; 2001. 325 p.

14. Dubois A, Gadde LE. Systematic combining: An abductive approach to case research. J Bus Res.

For peer review only

For peer review only - http://bmjopen.bmj.com/site/about/guidelines.xhtml

2002;55(7):553–60.

15. O'Brien BC, Harris IB, Beckman TJ, Reed DA, Cook DA. Standards for reporting qualitative research: A synthesis of recommendations. Acad Med. 2014;89(9):1245–51.

16. Spradley JP. Participant observation. New York: Holt, Rinehart and Winston; 1980. 195 p.

17. Arman R, Vie OE, Åsvoll H. Refining shadowing methods for studying managerial work. In: The Work of ManagersTowards a Practice Theory of Management [Internet]. Oxford University Press; 2012. p. 301–17. Available from: http://www.oxfordscholarship.com/view/10.1093/acprof:oso/9780199639724.001.0001/acprof-9780199639724-chapter-16

18. Strauss A, Corbin J. Basics Of Qualitative Research: Techniques And Procedures For Developing Grounded Theory. 4 th ed. London: SAGE Publications; 1998. 456 p.

19. Vicente KJ. Cognitive work analysis : toward safe, productive, and healthy computer-based work. London, England: Lawrence Erlbaum Associates; 1999. xix, 392 p.

20. Goldenhar LM, Brady PW, Sutcliffe KM, Muething SE. Huddling for high reliability and situation awareness. BMJ Qual Saf. 2013;22(11):899–906.

21. Gorman JC, Cooke NJ, Amazeen PG. Training adaptive teams. Hum Factors. 2010;52(2):295–307.

22. Ekstedt M, Cook RI. The stockholm blizzard of 2012. In: Resilient Health Care, Volume 2: The Resilience of Everyday Clinical Work. 2017. p. 59–74.

23. Weick KE, Sutcliffe KM. Managing the expected: Sustained performance in a complex world. 3rd ed. Wiley; 2015. 224 p.

24. Anderson JE, Ross AJ, Jaye P. Modelling Resilience and Researching the Gap between Work-as-

For peer review only

For peer review only - http://bmjopen.bmj.com/site/about/guidelines.xhtml

Imagined and Work-as-Done. In: J. Braithwaite, R. L. Wears RL and EH, editor. Resilient Health Care, Volume 3, Reconciling Work-as-Imagined and Work-as-Done. Farnham: Ashgate; 2017. p. 133–41.

25. Lincoln YS. Emerging Criteria for Quality in Qualitative and Interpretive Research. Qual Inq [Internet]. 1995 Sep 29;1(3):275–89. Available from: http://methods.sagepub.com/book/the-qualitative-inquiry-reader/n19.xml

26. Elo S, Kääriäinen M, Kanste O, Pölkki T, Utriainen K, Kyngäs H. Qualitative Content Analysis. SAGE Open. 2014;4(1):215824401452263.

27. Johnson B. Ethical issues in shadowing research. Qual Res Organ Manag An Int J [Internet]. 2014;9(1):21–40. Available from: http://www.emeraldinsight.com/doi/10.1108/QROM-09-2012-1099

28. Praetorius G, Hollnagel E, Dahlman J. Modelling Vessel Traffic Service to understand resilience in everyday operations. Reliab Eng Syst Saf. 2015;141:10–21.

Figure legends

Fig. 1 The studied NICU in a tertiary level hospital with surgical capacity. Managers are visually located to illustrate their vicinity to the clinical care teams. Overarching goals are presented at the base of the figure with examples of how they are expressed in daily work. A selection of peripheral wards and units are illustrated in the surrounding area. The arrows are double headed to symbolise a two-way relationship of demands and possibilities for negotiation of for example patient transfers.

Fig. 2 Illustration of the work of balancing goal-settings for system wide coherence, maintaining the umbrella perspective and streamlining communication to meet rapidly changing demands with the shadowed clinical coordinator at the centre. The width of double headed arrows visualises an estimation of the most frequently observed communication for the respective manager.

For peer review only

For peer review only - http://bmjopen.bmj.com/site/about/guidelines.xhtml

533

For peer review only

For peer review only - http://bmjopen.bmj.com/site/about/guidelines.xhtml

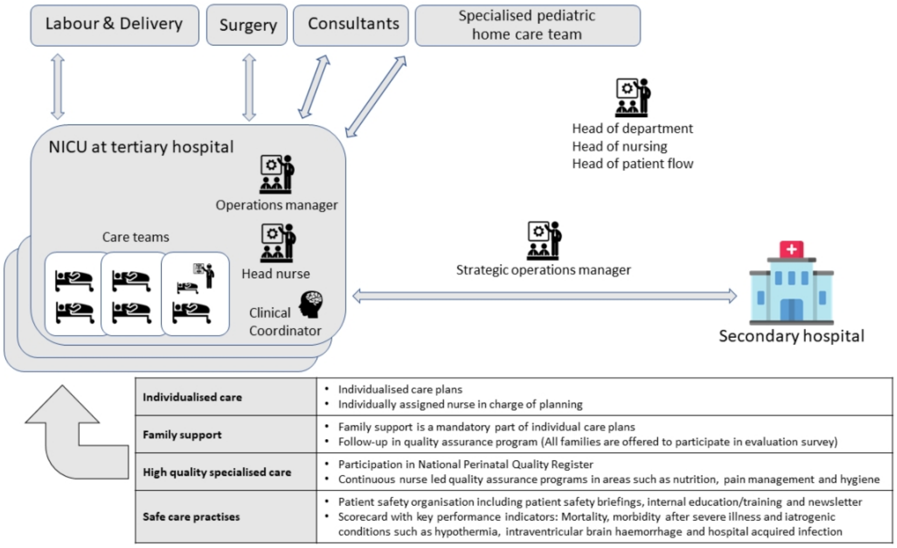

Fig. 1 The studied NICU in a tertiary level hospital with surgical capacity. Managers are visually located to illustrate their vicinity to the clinical care teams. Overarching goals are presented at the base of the figure with examples of how they are expressed in daily work. A selection of peripheral wards and units are illustrated in the surrounding area. The arrows are double headed to symbolise a two-way relationship of demands and possibilities for negotiation of for example patient transfers.

108x60mm (300 x 300 DPI)

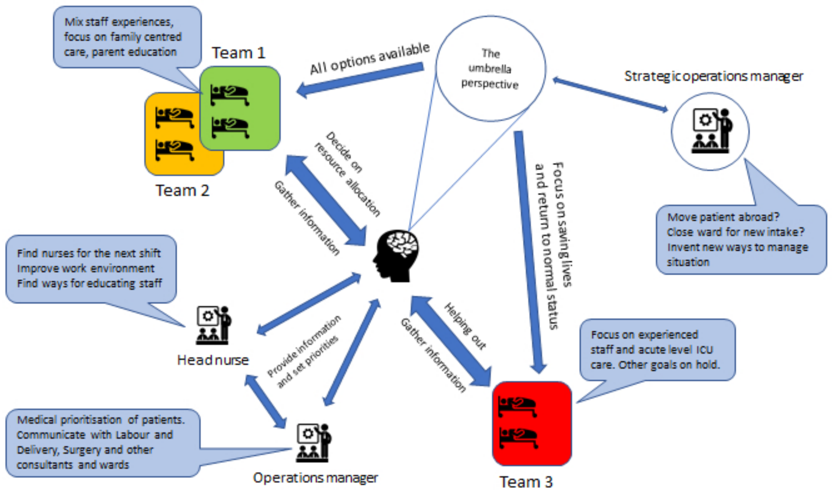

Fig. 2 Illustration of the work of balancing goal-settings for system wide coherence, maintaining the umbrella perspective and streamlining communication to meet rapidly changing demands with the shadowed clinical coordinator at the centre. The width of double headed arrows visualises an estimation of the most frequently observed communication for the respective manager.

67x38mm (300 x 300 DPI)

Appendix 1

Q1

(Clinical coordinator) We have put those two [patients] together in 9:1 to get an emergency cot in 9:2 for the twins [9 refers to a room divided in two sections equipped for one patient per section 9:1 and 9:2], I told the father that they may have to move out. But then we'll be in the situation where they maybe… They may need… That father is very new. So, I think that in that case they'll have to be two [staff] out there too.
(Operations manager) The [NN] twins?
(Head nurse) Right. Now there are two kids there, so one person can take care of them. But if you add those two [twins] then there'll be four babies, so then you need another person out there [on the ward] and there'll only be two staff left in there [room 9].
(Operations manager) Mm, it's tricky.
(Head nurse) Oh... so there are three of them who can help each other and… in [room] 7 and 8?
(Operations manager) Okay… I understand that to mean that we have a bit of leeway here if we need it in an emergency... but nothing that we can give to anyone else. Okay. [previously wanted to lend a nurse to another ward in need]
(Head nurse) Yeah, that's too bad. We'd love to be able to.
(Operations manager) Take over week 36 twins who need it, say. And then send them back, that's no fun.
(Clinical coordinator) And then we'd have to open like [rooms] 13 and 14.

Q2

The coordinator calls for an ad-hoc on-site face-to-face discussion with the strategic operations manager. This happens when the CMT experience that basic safe care practices at the clinical level consume every opportunity for maintaining overarching quality goals, the unit stop lending help to others and start sacrificing continuity of care for individual patients. The plan for today is to admit one intermediary level patient that were born during the night and is waiting for a room at the NICU. One patient has been diagnosed with a multi-resistant bacterium and will need increased hygiene standards. One baby currently in another hospital are being assessed by a surgeon for the need of possible transport and surgery, one will possibly arrive by helicopter for eye surgery today. Three babies are planned for being transported back to their respective local hospitals, one by helicopter today and twins tomorrow (Observers notes after the coordinating management team's morning huddle).

- *(Clinical coordinator) We are plenty of people today, that is nice. But when NN [strategic operations manager] asked if we had a lot of capacity, I had to say no.*

The clinical coordinator checks the antenatal list of at-risk mothers who are under observation.

- *(Clinical coordinator) Oh, there is a lot here, induction, ich... a lot of bleedings. There is a lot waiting... but you don't know when... It could be calm... I will not read all their charts.*
- *(Operations manager) ...one important thing. This baby that needed eye surgery is now acute and will arrive soon. They will land directly in the operating room and the transport team will take care of the baby until it can go back home its own hospital.*
- *(Strategic operations manager) Can we lend out some staff to [a neonatal ward in a neighbouring hospital].*
- *(Head nurse) I can check, we have plenty of staff, but they are all in use.*

For peer review only

- *(Strategic operations manager) I ask because they are not doing so well [the other unit]. Twins are being born and they are asking for allowance and resources to move them to another region.*

Q3

- *(Clinical coordinator) This is not good, its full [the ward]. We have no space when this eye baby arrives.*

The clinical coordinator walks to the room where a nurse oversees the twins that were supposed to be transported out the next day but are now showing symptoms of infection.

- *(Clinical coordinator) Lets´ see when [Strategic operations manager] gets back, maybe we can send someone else.*
- *(Nurse) But they went to fetch the week 31 from delivery to there [points to an empty cot].*
- *(Clinical coordinator) Yes, and the eye baby is coming [points toward the 2nd empty place for a cot. Then its full here. Do you think these twins could be together in a twin cot?*
- *(Nurse) Well, I don´t know. This one is just on the margin of managing without incubator, and that one is getting treatment for bilirubin [treatment includes being exposed to light from a special lamp].*
- *(Clinical coordinator) The problem is that I don't have staff to open another room.*
- *(Nurse) Maybe if we reconsider the lamp-treatment...*
- *(Clinical coordinator) Exactly, and then we hope that the eye baby can go back to his own hospital later in the evening.*

For peer review only

Standards for Reporting Qualitative Research (SRQR)

O'Brien B.C., Harris, I.B., Beckman, T.J., Reed, D.A., & Cook, D.A. (2014). Standards for reporting qualitative research: a synthesis of recommendations. *Academic Medicine, 89(9)*, 1245-1251.

**No. Topic**	**Item**
**Title and abstract**	
S1 Title	Concise description of the nature and topic of the study identifying the study as qualitative or indicating the approach (e.g., ethnography, grounded theory) or data collection methods (e.g., interview, focus group) is recommended<br><br>**Qualitative descriptive study is part of the title.**
S2 Abstract	Summary of key elements of the study using the abstract format of the intended publication; typically includes objective, methods, results, and conclusions<br><br>**The 300 words abstract contain objectives, design, participants, results and conclusions**
**Introduction**	
S3 Problem formulation	Description and significance of the problem/phenomenon studied; review of relevant theory and empirical work; problem statement<br><br>**Problem statement on line 68-71 followed by relevant theory. Study Aim is located at line 90-92.**
S4 Purpose or research question	Purpose of the study and specific objectives or questions<br><br>**The explorative qualitative design is guided by the study aim. A specific area for improvement in indicated on line 88-89.**
**Methods**	
S5 Qualitative approach and research paradigm	Qualitative approach (e.g., ethnography, grounded theory, case study, phenomenology, narrative research) and guiding theory if appropriate; identifying the research paradigm (e.g., positivist, constructivist/interpretivist) is also recommended<br><br>**The ethnographic design and participatory observations are described in the methods section, sub headline "Design" line 95-99.**
S6 Researcher characteristics and reflexivity	Researchers' characteristics that may influence the research, including personal attributes, qualifications/experience, relationship with participants, assumptions, or presuppositions; potential or actual interaction between researchers' characteristics and the research questions, approach, methods, results, or transferability

For peer review only

	**The researchers characteristics is mentioned in the methodological bulletpoint on page 3 line 63-64 and further discussed in "Limitations" line 403-405. Trustworthiness is discussed in the second last paragraph in "Limitations"**
S7 Context	Setting/site and salient contextual factors; rationale[a]<br><br>**Setting is presented in the *introductions first paragraph* and a subheading under methods "Design and setting" Line 100-105.**
S8 Sampling strategy	How and why research participants, documents, or events were selected; criteria for deciding when no further sampling was necessary (e.g., sampling saturation); rationale[a]<br><br>**The sample of data are mentioned in line 119-127. Saturation is not specifically discussed.**
S9 Ethical issues pertaining to human subjects	Documentation of approval by an appropriate ethics review board and participant consent, or explanation for lack thereof; other confidentiality and data security issues.<br><br>**Ethics approval information on line 436.**
S10 Data collection methods	Types of data collected; details of data collection procedures including (as appropriate) start and stop dates of data collection and analysis, iterative process, triangulation of sources/methods, and modification of procedures in response to evolving study findings; rationale[a]<br><br>**Data collection and data sources are presented in the methods section, sub-headline "Data collection".**
S11 Data collection instruments and technologies	Description of instruments (e.g., interview guides, questionnaires) and devices (e.g., audio recorders) used for data collection; if/how the instrument(s) changed over the course of the study<br><br>**Audio recording presented on line 137. Other instruments are not applicable in the ethnographic design.**
S12 Units of study	Number and relevant characteristics of participants, documents, or events included in the study; level of participation (could be reported in results)<br><br>**Not applicable in the ethnographic design, ample data is presented through quotes and appendix 1.**
S13 Data processing	Methods for processing data prior to and during analysis, including transcription, data entry, data management and security, verification of data integrity, data coding, and anonymization/deidentification of excerpts<br><br>**Analysis and collection of data were iterated according to the study design.**
S14 Data analysis	Process by which inferences, themes, etc., were identified and developed, including researchers involved in data analysis; usually references a specific paradigm or approach; rationale[a]<br><br>**Analysis method presented in line 141-152 and "table 1" for further transparency.**
S15 Techniques to enhance trustworthiness	Techniques to enhance trustworthiness and credibility of data analysis (e.g., member checking, audit trail, triangulation); rationale[a]

For peer review only

	**Trustworthiness discussed in line 405-411**
**Results/Findings**	
S16 Synthesis and interpretation	Main findings (e.g., interpretations, inferences, and themes); might include development of a theory or model, or integration with prior research or theory<br><br>**Results is based on an interpretation through a lens of the theoretical concept of resilience. Integration with theory is found in the discussion.**
S17 Links to empirical data	Evidence (e.g., quotes, field notes, text excerpts, photographs) to substantiate analytic findings<br><br>**Extensive quotes and field notes presented in results and appendix 1.**
**Discussion**	
S18 Integration with prior work, implications, transferability, and contribution(s) to the field	Short summary of main findings; explanation of how findings and conclusions connect to, support, elaborate on, or challenge conclusions of earlier scholarship; discussion of scope of application/generalizability; identification of unique contribution(s) to scholarship in a discipline or field<br><br>**Integration with existing management theory for example in line 328-331, 358-360, 372-373.**
S19 Limitations	Trustworthiness and limitations of findings<br><br>**Trustworthiness and limitations are discussed in lines 390-416**
**Other**	
S20 Conflicts of interest	Potential sources of influence or perceived influence on study conduct and conclusions; how these were managed<br><br>**No competing interests line 445**
S21 Funding	Sources of funding and other support; role of funders in data collection, interpretation, and reporting<br><br>**No specific funding line 447.**

[a]The rationale should briefly discuss the justification for choosing that theory, approach, method, or technique rather than other options available, the assumptions and limitations implicit in those choices, and how those choices influence study conclusions and transferability. As appropriate, the rationale for several items might be discussed together

For peer review only
